# Supplementary material for: Cell-type-specific DNA methylation dynamics in the prenatal and postnatal human cortex
Source: Cell Genom. 2025 Sep 24;5(12):101010. doi: 10.1016/j.xgen.2025.101010 (PMC12802690; doi:10.1016/j.xgen.2025.101010)
Supplement: Document S1. Figures S1–S29 [file mmc1.pdf]

Cell Genomics, Volume *5*

Supplemental information

Cell-type-specific DNA methylation dynamics

in the prenatal and postnatal human cortex

Alice Franklin, Jonathan P. Davies, Nicholas E. Clifton, Georgina E.T. Blake, Rosemary Bamford, Emma M. Walker, Barry Chioza, Martyn Frith, APEX Consortium, Youth-GEMs Consortium, Joe Burrage, Nick Owens, Shyam Prabhakar, Emma Dempster, Eilis Hannon, and Jonathan Mill

Supplementary Figures

Figure S1 - Age estimates derived from an epigenetic clock calibrated on fetal brain are correlated with gestational age in fetal cortex, related to STAR Methods. Across the 91 fetal cortex samples used in this study there was a strong correlation between actual age and estimated gestational age derived from a fetal brain epigenetic clock (corr = 0.942) [1]. Shaded region indicates 95% confidence interval.

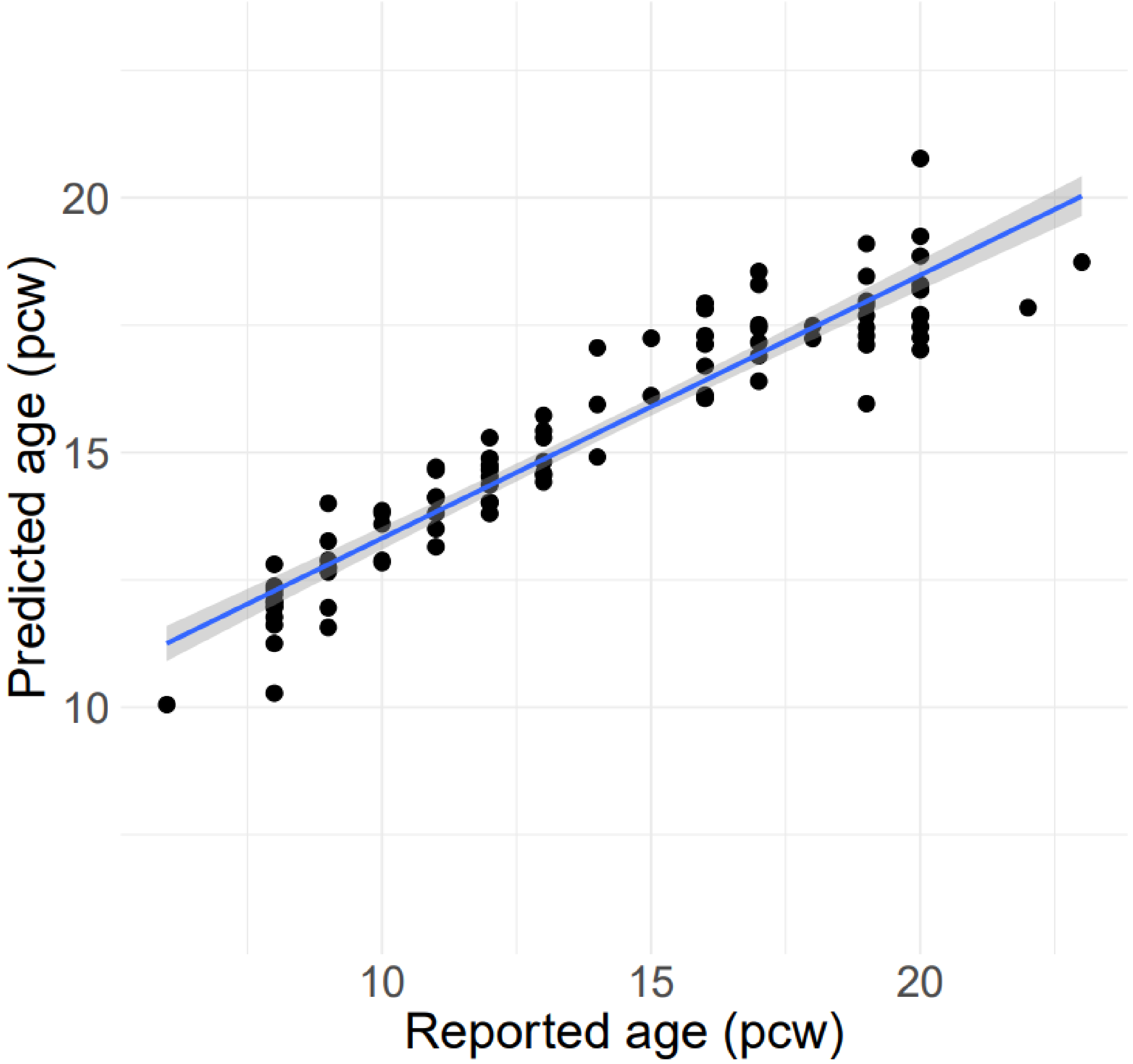

Figure S2 - Developmental changes in DNA methylation in the human cortex are highly correlated with those reported in two previous studies of the developing fetal brain performed using older technology, related to Figure 1. Effect sizes for dDMPs overlapping with dDMPs reported in **A)** Spiers *et al.* [2] (number of overlapping sites = 20,502, corr = 0.824, $p < 1x10^{-320}$) and **B)** Numata *et al.* [3] (number of overlapping sites = 66, corr = 0.94, $p = 2.8 \times 10^{-31}$). Dotted line = y=x.

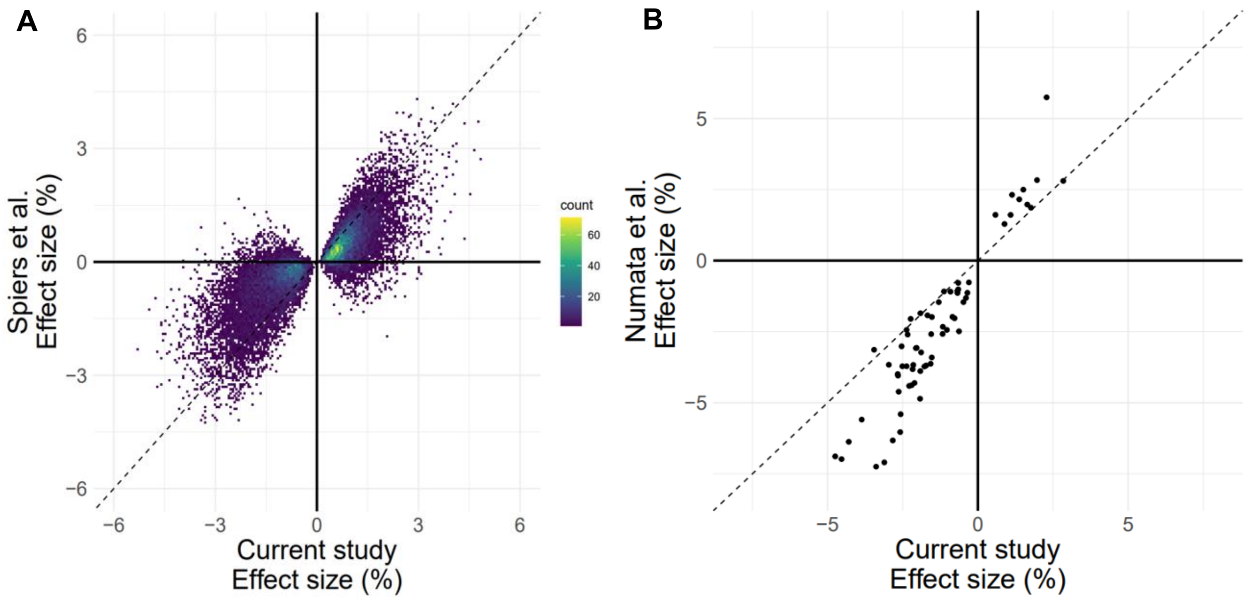

Figure S3 - Distribution of effect sizes for the 50,913 dDMPs, split by direction of effect, related to Figure 1. The mean effect size (absolute change in DNA methylation (%) per week) for hypermethylated dDMPs (red) is smaller than for hypomethylated dDMPs (blue) ($p < 1x10^{-320}$). Dotted lines indicate the mean absolute effect sizes for hypermethylated and hypomethylated dDMPs.

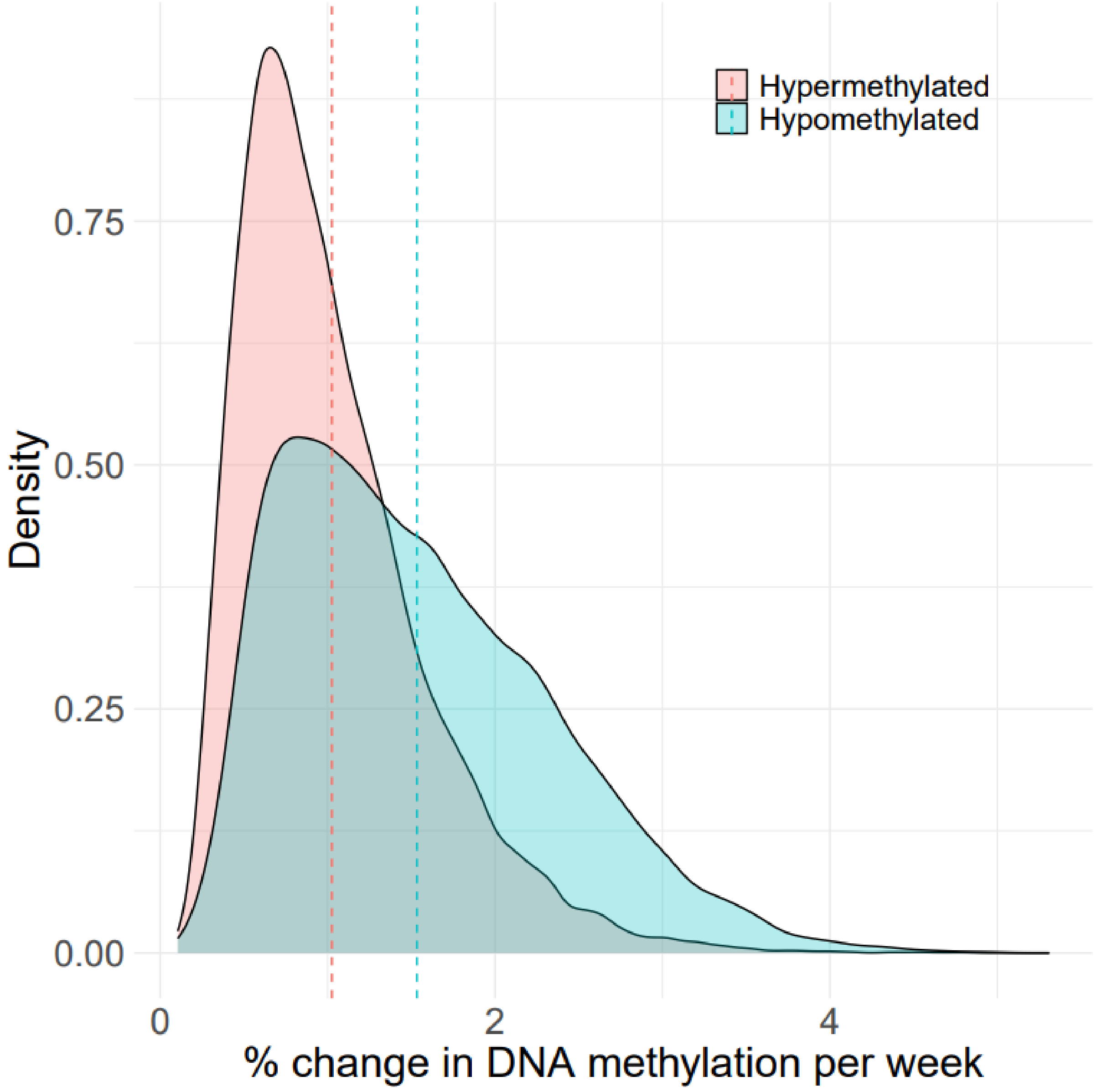

Figure S4 - Mean global DNA methylation decreases across autosomal sites during cortex development, related to Figure 1. A) Across all autosomal sites tested (n = 790,180), the mean level of DNA methylation decreases with fetal age (% change in DNA methylation per week = -0.0194, p = 3.33×10^{-10}). **B)** The same data is presented as in **A** but the y-axis is scaled from 0 - 100% DNA methylation highlighting the very small magnitude of change across this period. **C)** After excluding autosomal fetal cortex dDMPs, mean global DNA methylation across the remaining 739,839 autosomal sites significantly increases during cortex development (% change in DNA methylation per week = 0.00794, p = 9.74×10^{-3}). **D)** The same data is presented as in **C)** but the y-axis is scaled from 0 - 100% DNA methylation.

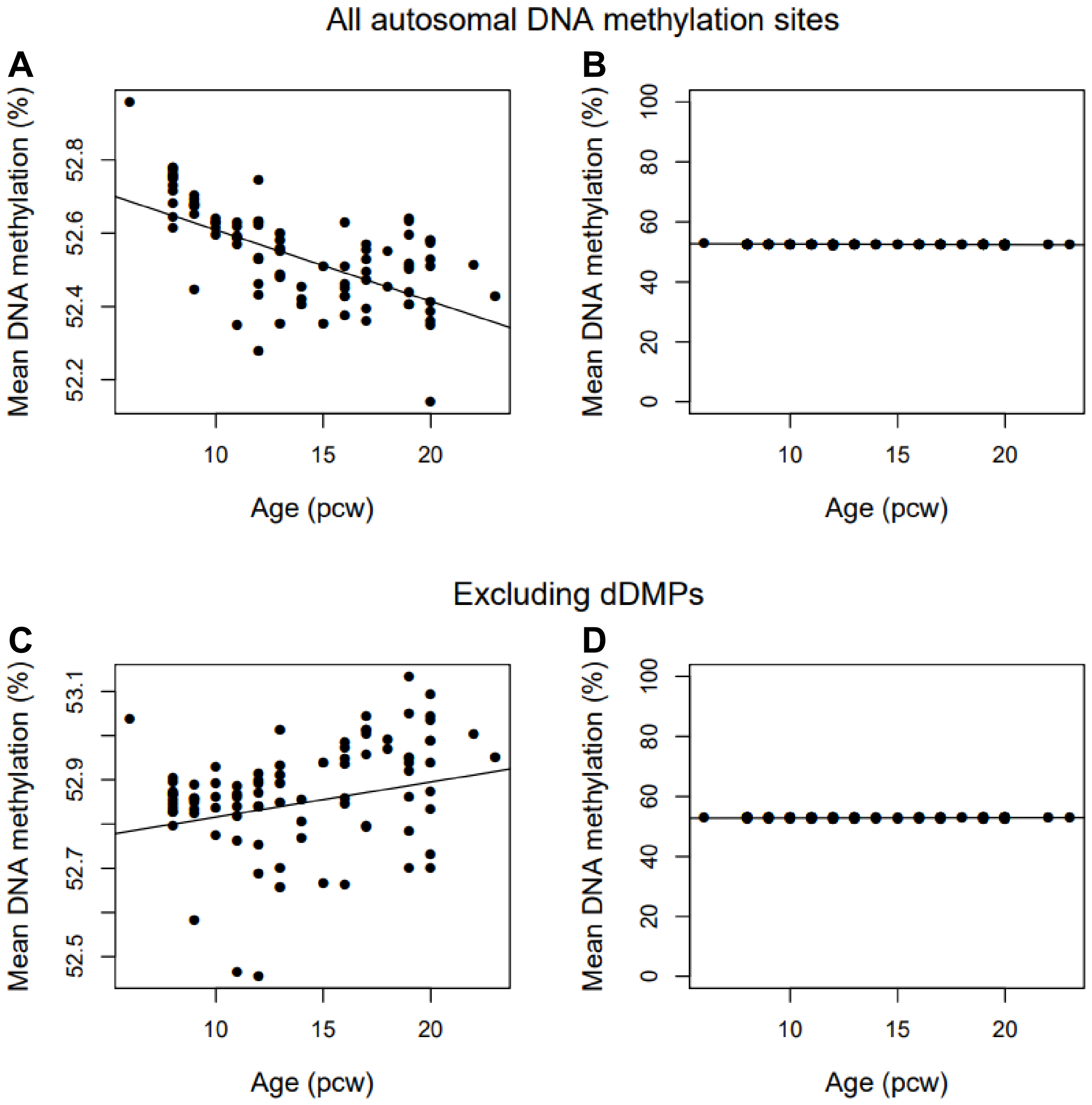

Figure S5 – A large proportion of dDMPs are characterized by nonlinear changes in DNA methylation, related to Figure 2. A) Overlap of the 50,913 dDMPs ("Linear dDMPs") identified using a linear regression model and the 73,035 sites classified as showing nonlinear changes in DNA methylation across development using Gaussian process modelling ("Nonlinear sites"). **B)** cg01848383 (annotated to *FOXP1*, a high-confidence SFARI autism gene) is an example of a hypermethylated nonlinear-dDMP and **C)** cg04292453 (annotated to *TRIO*, another high-confidence SFARI autism gene) is an example of a hypomethylated nonlinear-dDMP. A full list of sites at which DNA methylation changes non-linearly across cortex development is given in **Table S4**.

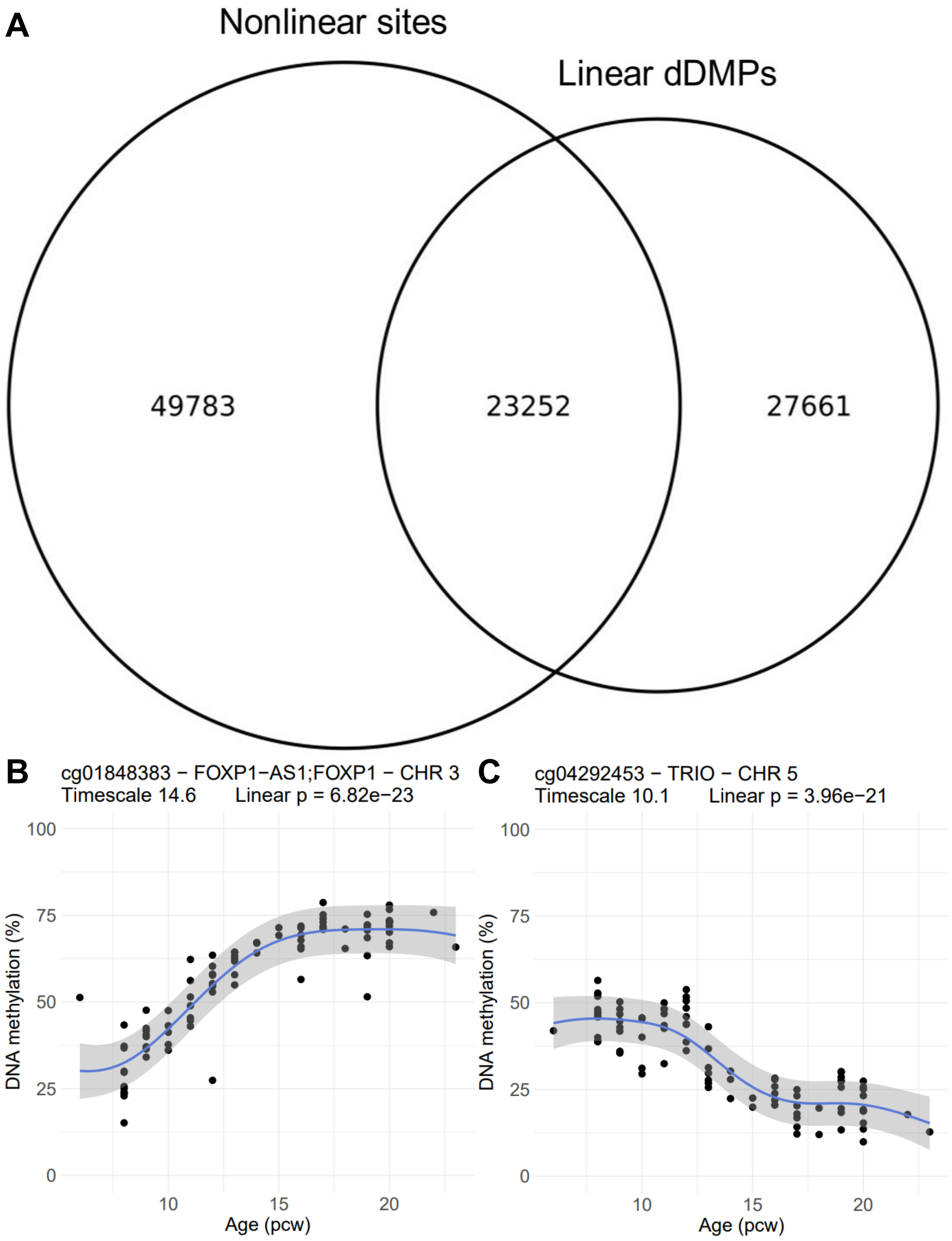

Figure S6 - The distribution of dDMPs across chromosomes was relatively consistent apart from a notable depletion on chromosome 19, related to Figure 1. A) Certain chromosomes were characterized by a significant enrichment or depletion of dDMPs (see also **Table S6**). Red = significant enrichment of dDMPs, blue = significant depletion of dDMPs, grey = no significant enrichment or depletion.

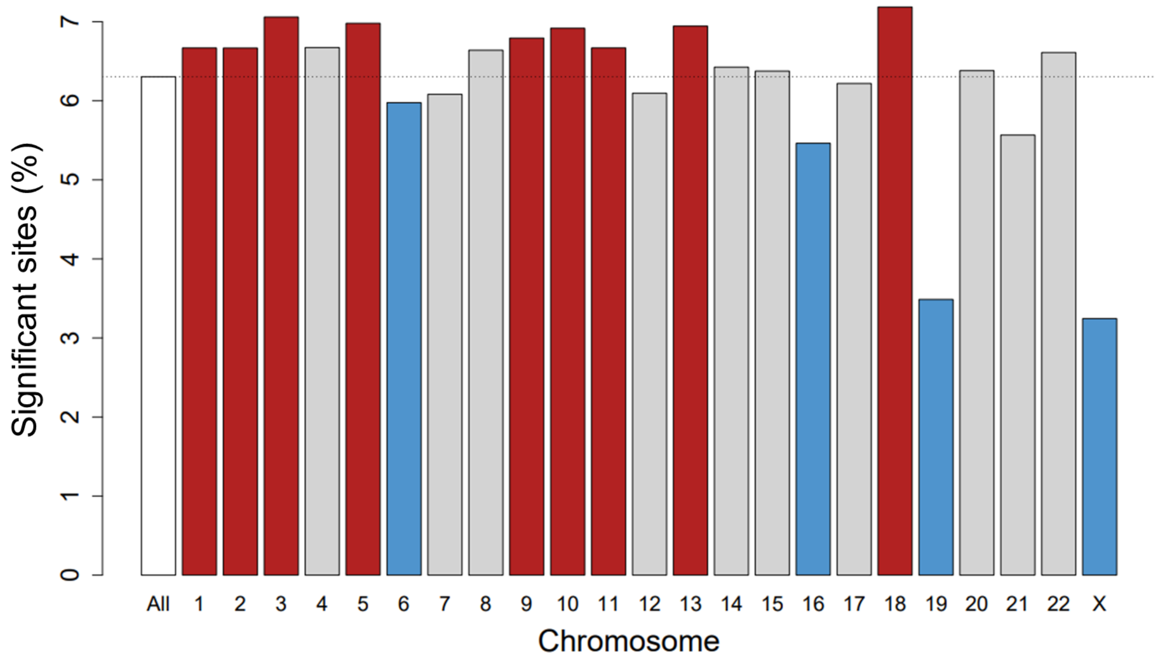

Figure S7 – Cortex dDMPs are enriched in regions of open chromatin identified using scATAC-seq, related to STAR Methods and Figure 1. A) Relative effect size from a logistic regression analysis testing for an enrichment of linear dDMPs within scATAC-seq peaks obtained from a published analysis of 54 human fetal cell-types [4]. 31 out of the 54 cell-types tested were characterized by significant enrichment, with the strongest effect being found for excitatory neurons. The color indicates the direction of effect (red = significant enrichment, blue = significant depletion, gray = non-significant). Full cell-type labels (x-axis) can be found in **Table S16**. **B)** Heatmap showing relative enrichment within cell-type-specific ATAC-seq peaks of all linear and non-linear dDMPs (left) and non-linear sites within individual WCGNA modules (right).

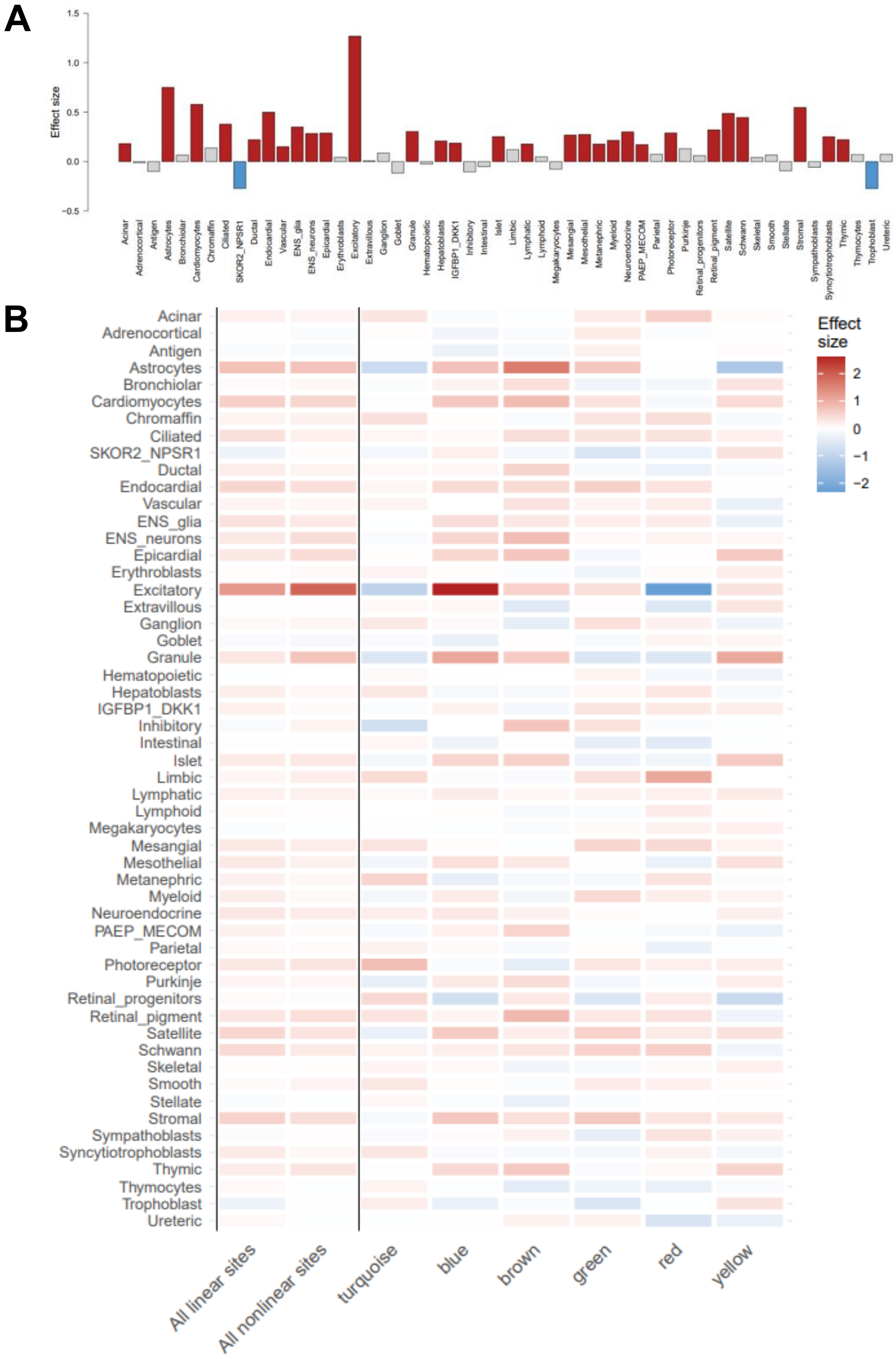

Figure S8 - Age estimates derived from epigenetic clocks calibrated on postnatal samples are strongly correlated with the reported age for the postnatal cortex samples included in this study, related to STAR Methods. Across the 677 late-fetal and postnatal bulk cortex samples included in this study (26pcw - 104 years), estimated age was highly correlated to reported age using both **A)** a pan-tissue epigenetic clock (corr = 0.875) [5] and **B)** an epigenetic clock trained on postnatal cortex tissue (corr = 0.941) [6].

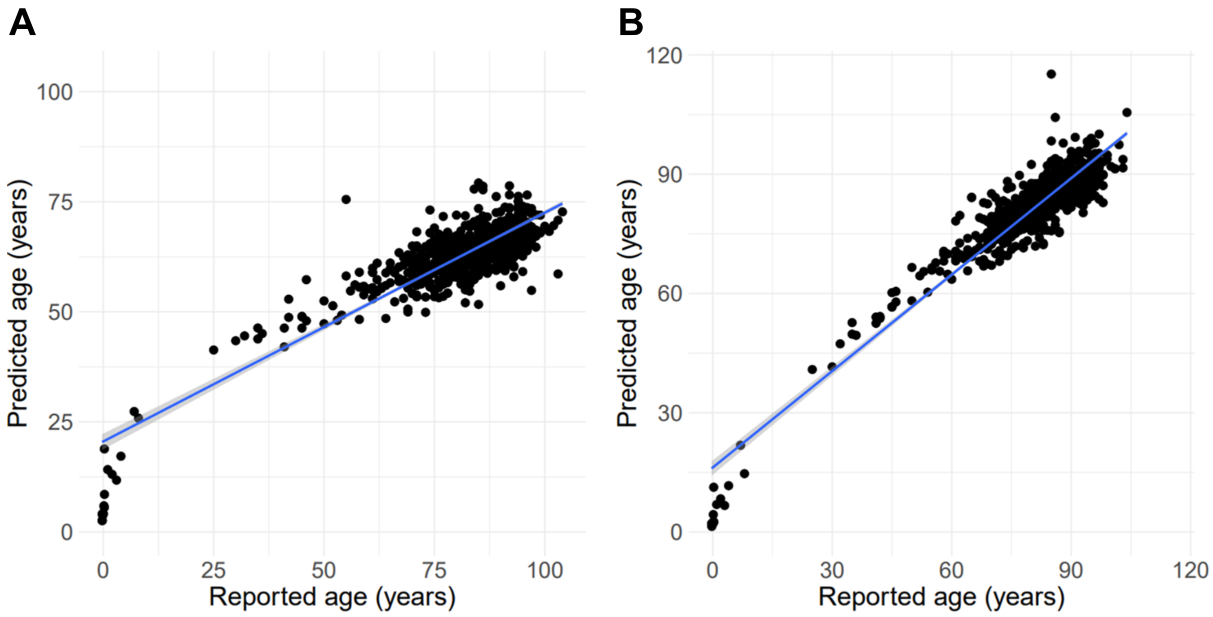

Figure S9 – Comparison of age-associated DNA methylation changes between prenatal and postnatal cortex across CpG island-related features, related to Figure 3. Plotted are effect sizes for 41,518 differentially methylated positions (dDMPs) identified in the prenatal cortex that were also present in our postnatal dataset. Each point represents a dDMP; points are colored according to whether the site was significantly associated with age in the postnatal cortex (gray = not significant). Significance thresholds were determined separately for each CpG island feature category, adjusted for the number of sites tested within each group. Overall, there was limited correlation between age effects in the prenatal and postnatal cortex, consistent with the genome-wide pattern observed in **Figure 3B**. However, certain genomic features, particularly CpG islands, exhibited more consistent methylation trajectories across development: approximately 12% of prenatal dDMPs located within CpG islands also showed significant age-associated methylation changes in the same direction postnatally.

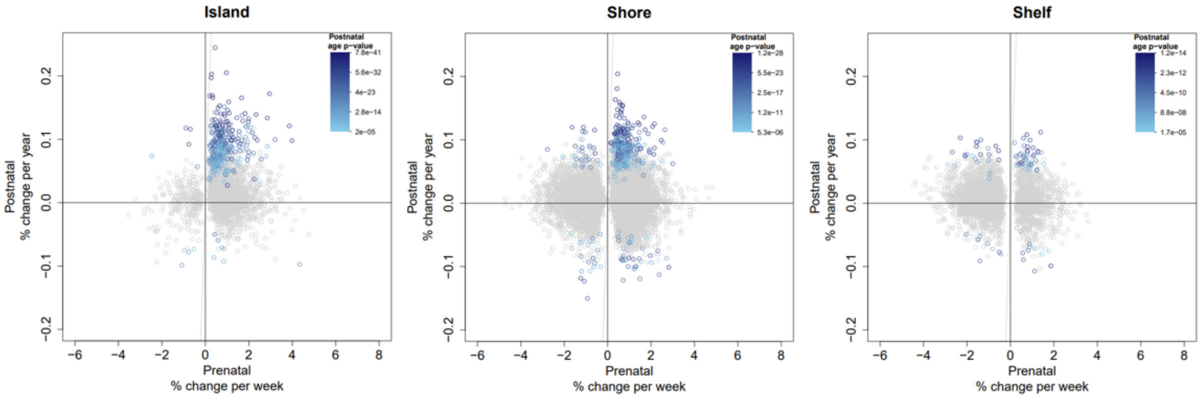

Figure S10 – Enrichment of sites associated with prenatal cortex development and postnatal age within different CpG island and genic features, related to Figure 3. A) As in **Figure 1D**, feature enrichment analysis of prenatal development-associated DMPs (dDMPs). These DMPs are significantly depleted in CpG islands (1.65% annotated to CGIs, $p < 1x10^{-320}$). **B)** Among the subset of prenatal dDMPs that are also significantly associated with age in postnatal cortex (n = 1,003), there is a significant enrichment in CGIs (8.86% annotated to CGIs, $p = 3.97x10^{-99}$).

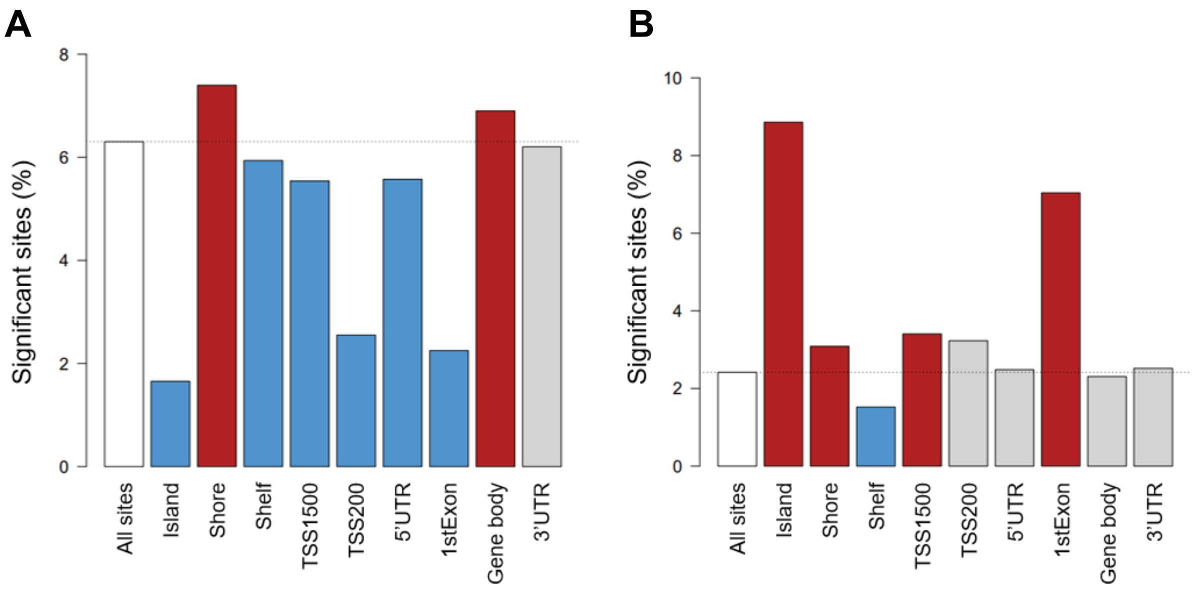

Figure S11 – Life-course trajectories of DNA methylation for the top-ranked hyper- and hypo-methylated dDMPs highlight development-specific effects, related to Figure 1 and Figure 3. The top hyper- and hypo-methylated dDMPs: cg08125539 (annotated to *IGF2BP1*, postnatal change in DNA methylation (%) per year = -0.0361, p = 0.290) and cg11884704 (annotated to *SLC25A25*, postnatal change in DNA methylation (%) per year = -0.0162, p = 0.493) show little variation in DNA methylation level after birth. pcw = post-conception weeks. yrs = years.

A

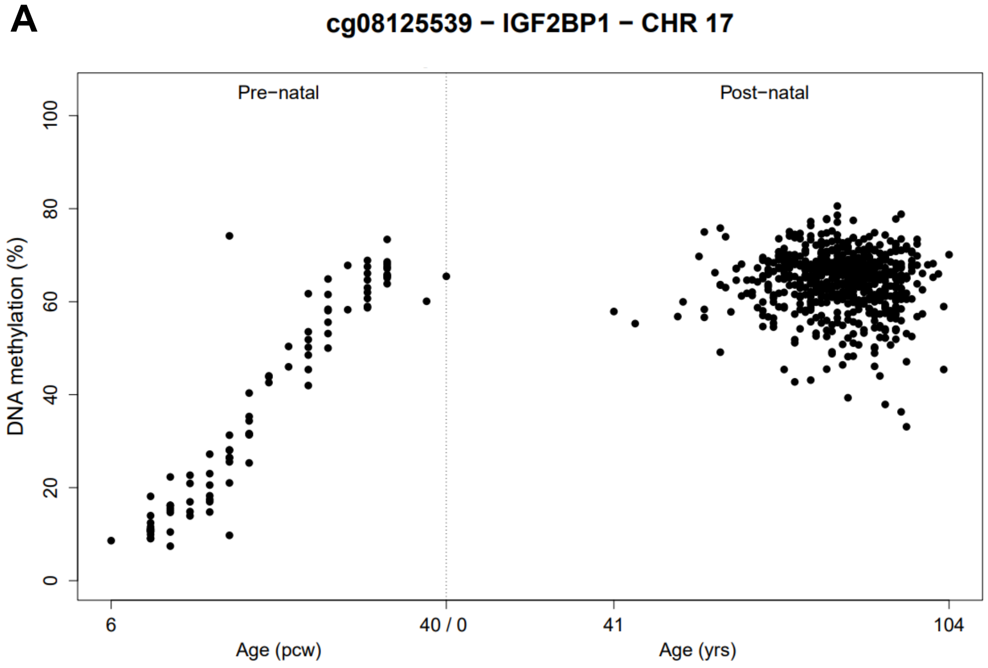

B

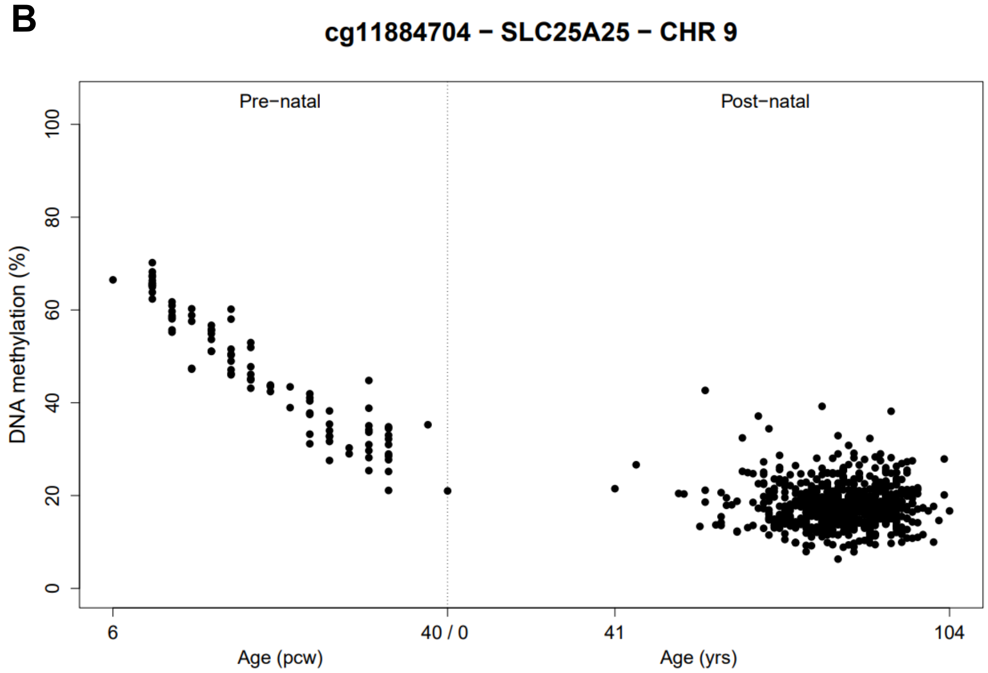

Figure S12 – The expression of *SATB2* is significantly higher than *RBFOX3* (encoding NeuN) during early- and mid-fetal cortex development, related to STAR Methods. Shown is expression data for *SATB2* and *RBFOX3* from the Brainseq Consortium [7] reported as reads per kilobase per million (RPKM) with standard error shown via shaded area. Developmental stages are defined as follows: Early Midfetal (12 - 18 pcw, n = 23), Midfetal (18 - 19 pcw, n = 14), Late Midfetal (19 - 26 pcw, n = 13), Late Fetal (26 - 40 pcw, n = 3), Early Infancy (0 - 180 days, n = 16), Early Childhood (1 - 6 years, n = 12), Late Childhood (6 - 13 years, n = 4), Adolescence (13 - 20 years, n = 47), Young Adulthood (20 - 30 years, n = 35), Mid Adulthood (30 - 60 years, n = 139), Late Adulthood (≥ 60 years, n = 28). Expression of *RBFOX3* was significantly lower than expression of *SATB2* in prenatal cortex ($t = -14.562$, $p < 2.2e-16$) but higher than expression of *SATB2* in postnatal cortex ($t = 25.01$, $p < 2.2e-16$).

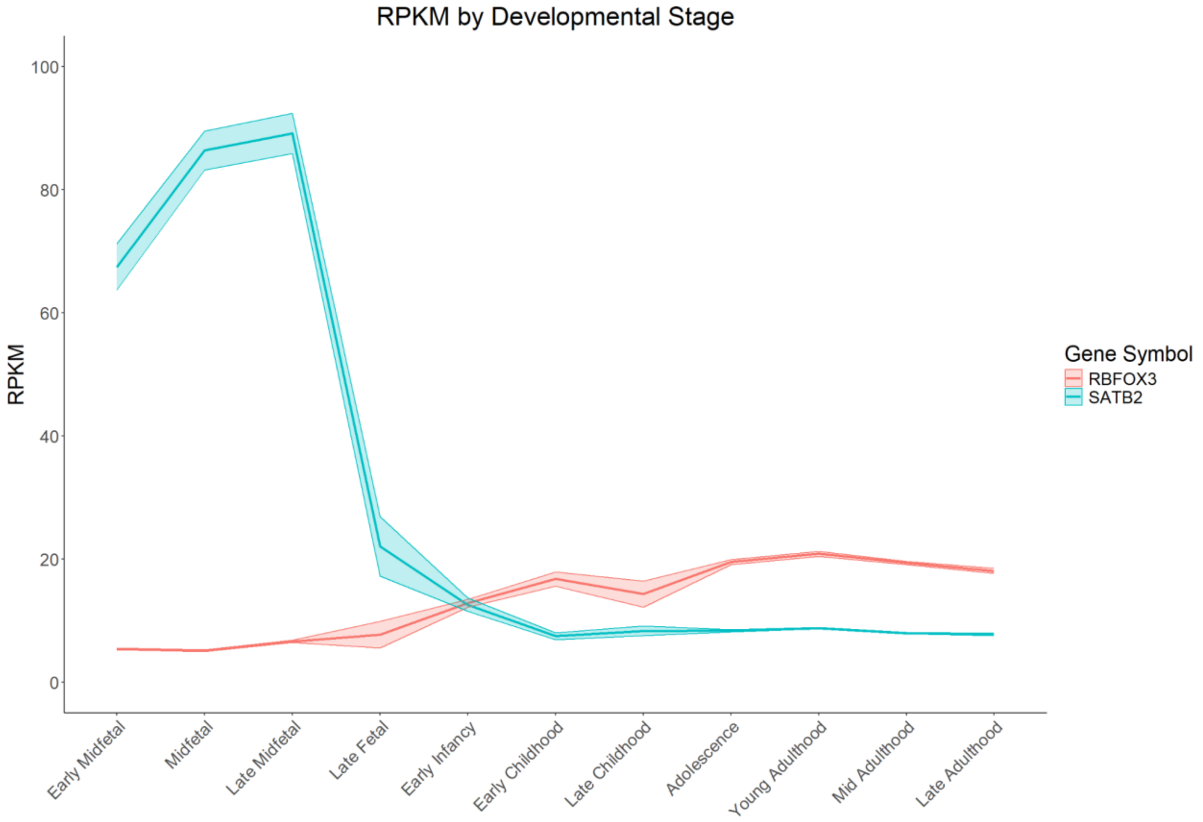

Figure S13 – Broad neuronal co-expression of *RBFOX3* and *SATB2* in postnatal cortex neurons, related to STAR Methods. Shown for total nuclei (bulk cortex) and FANS-isolated SATB2+ and NeuN+ nuclei is the expression of *SATB2* and *RBFOX3* across annotated cell-types quantified using single nuclei RNA-seq.

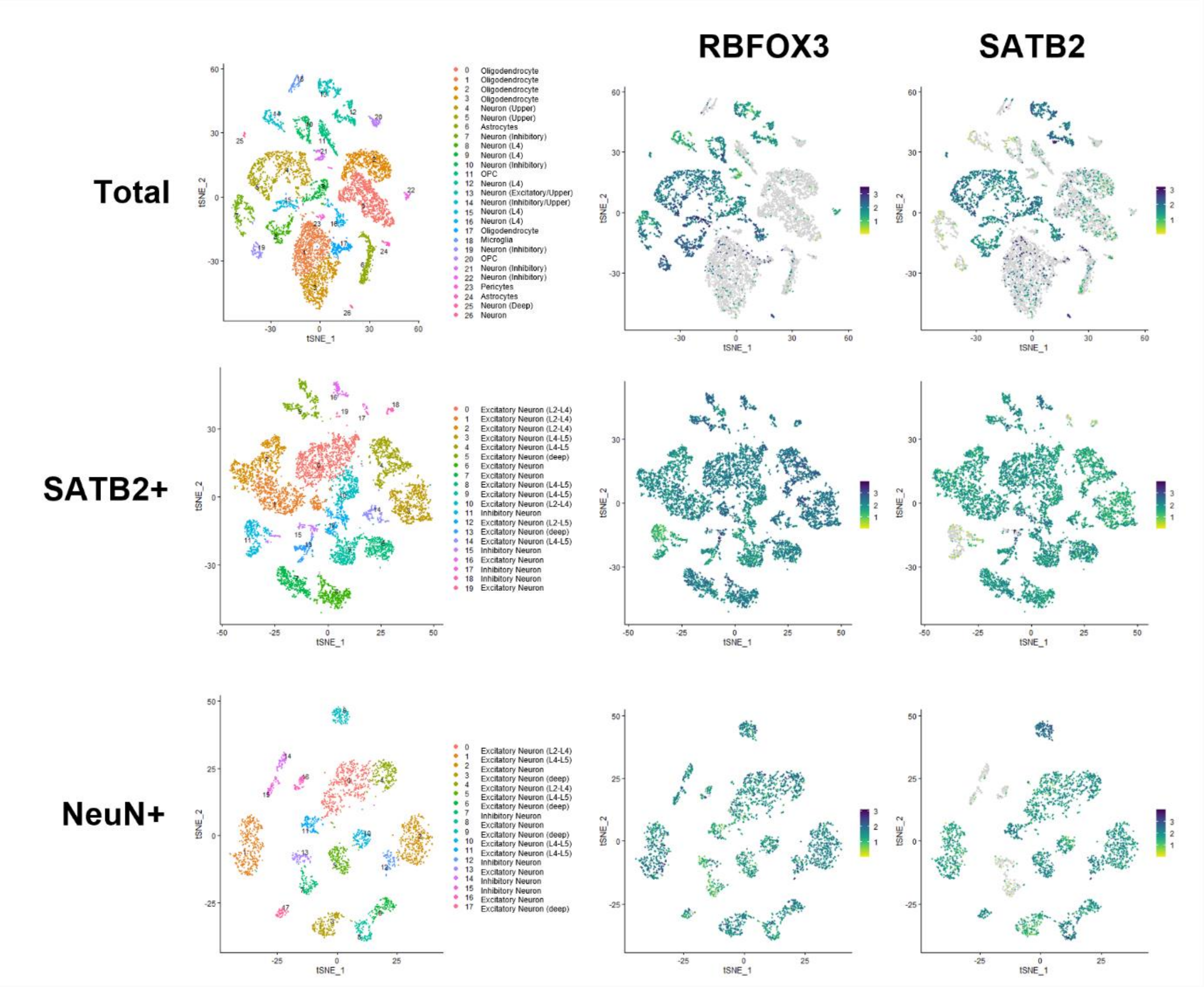

Figure S14 – *SATB2* expression is higher than *RBFOX3* expression in SATB2+ nuclei isolated from fetal cortex, related to STAR Methods. Shown for total nuclei (bulk cortex) and FANS-isolated SATB2+ nuclei is the expression of *SATB2* and *RBFOX3* across annotated cell-types quantified using snRNA-seq. The higher expression of *SATB2* in the fetal cortex parallels the data shown in **Figure S13**.

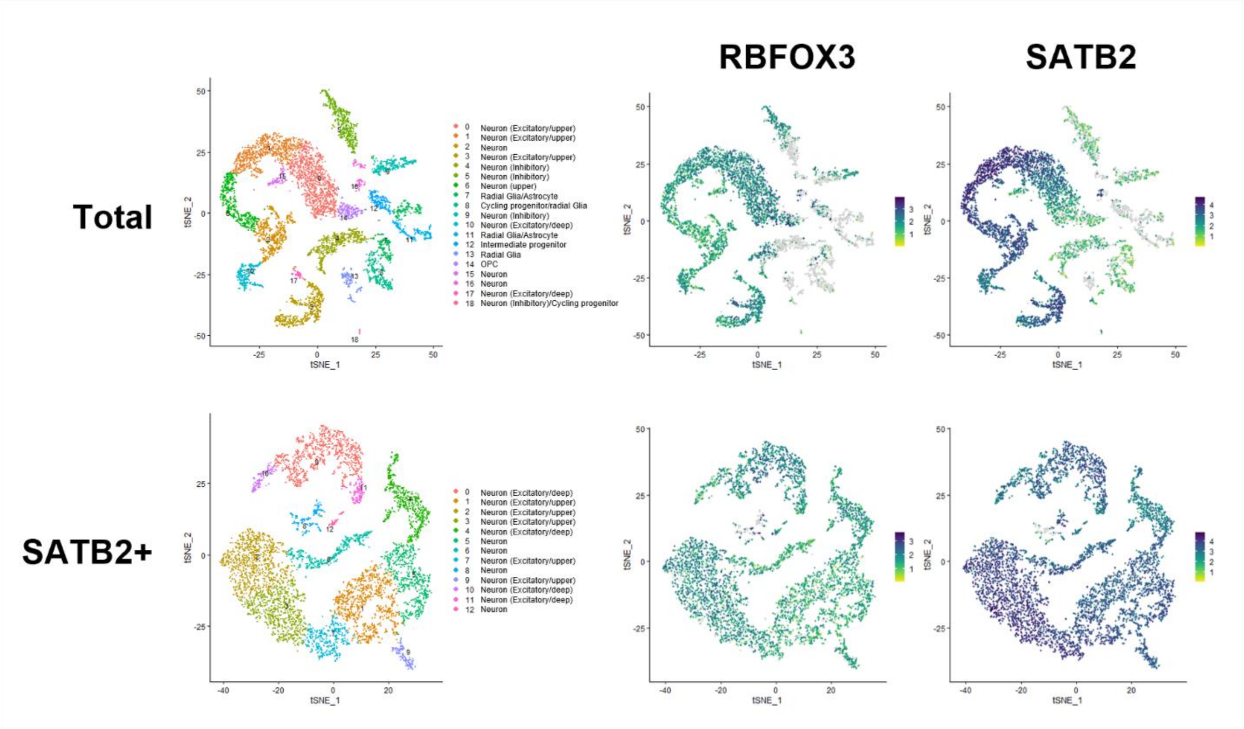

Figure S15 – A comparison of DNA methylation differences between neuron-enriched and neuron-depleted nuclei isolated from fetal and adult cortex, related to Figure 4. Scatter plots of NeuN+ (neuron-enriched) vs NeuN- (neuron-depleted) DNA methylation differences in adult cortex (n = 212 donors, x-axis) and SATB2+ (neuron-enriched) vs SATB2- (neuron-depleted) DNA methylation differences in fetal cortex (n = 37 donors, y-axis) for **A)** all sites tested in both (n = 693,895), **B)** sites identified as showing significant SATB2+ vs SATB2- differences in fetal cortex (n = 6,531) and **C)** sites identified as showing significant NeuN+ vs NeuN- differences in adult cortex (n = 453,675). Effect size represents the difference in mean DNA methylation (%) between nuclei populations. 'Count' in **A** represents the number of DNA methylation sites per bin.

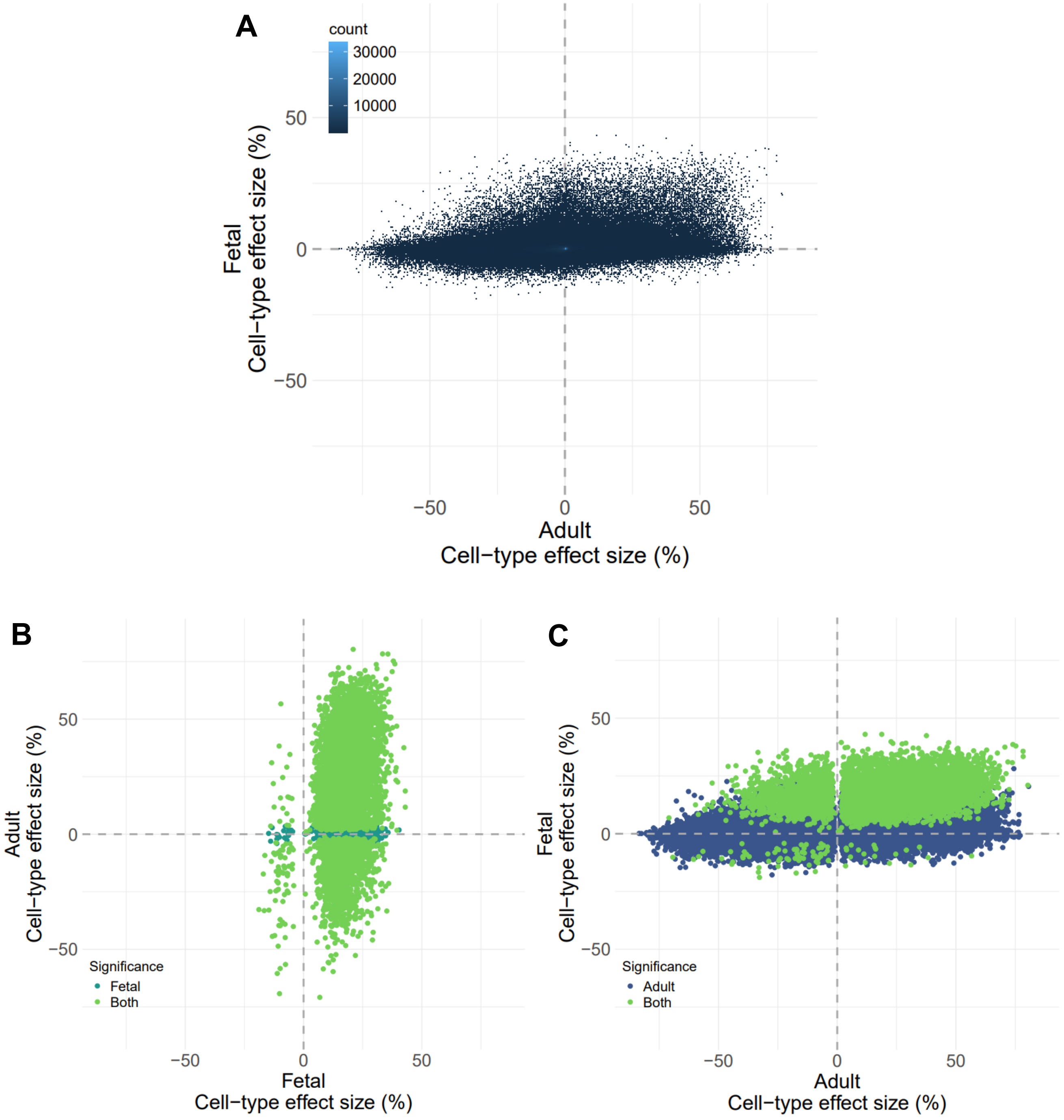

Figure S16 – Predicted cell-type proportions across early- and mid-fetal cortex samples derived using reference DNA methylation data, related to STAR Methods. ES = embryonic stem cells (ES) [8]. ES_NPC = ES-derived neural precursor cells [8]. SATB2pos = SATB2+ late-fetal (20 – 28pcw) and early postnatal (0 – 8y) samples (this study). NeuNpos = postnatal NeuN+ samples (this study). NeuNneg = postnatal NeuN- samples (this study). Shaded region indicates 95% confidence interval.

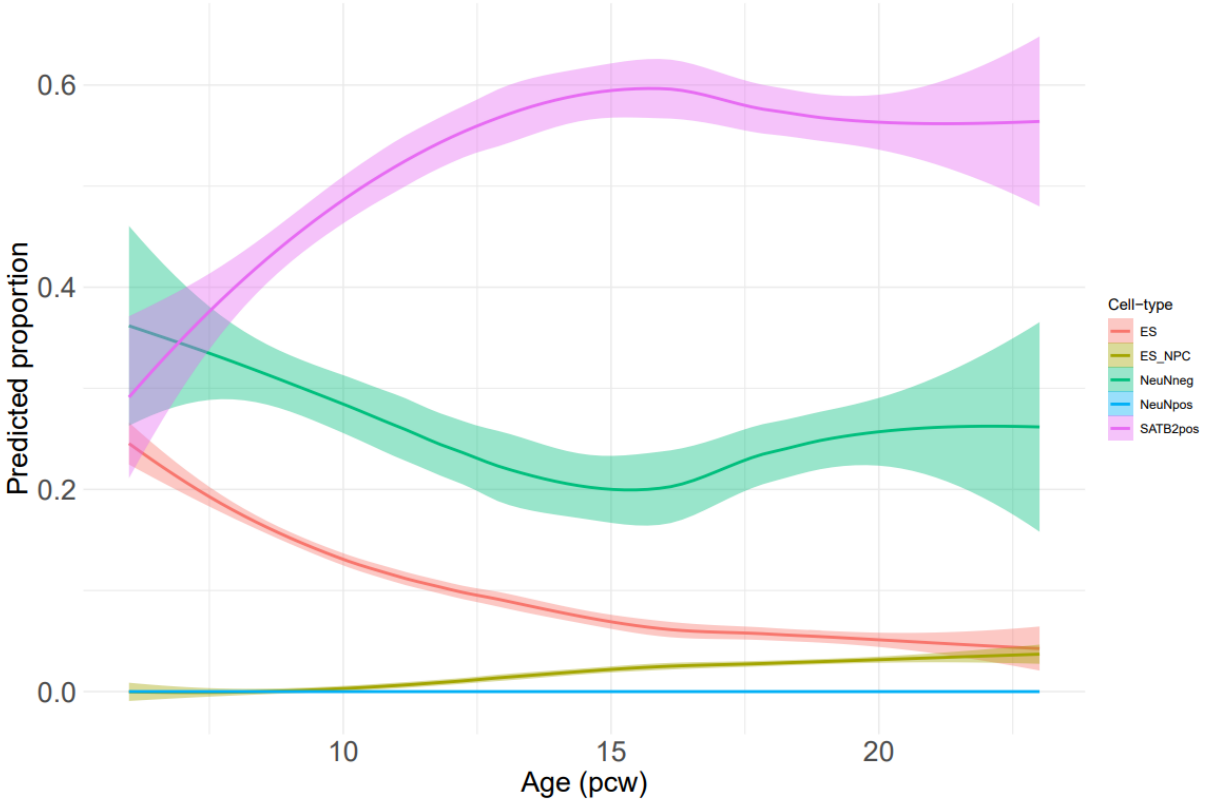

Figure S17 – Comparison of developmental changes in DNA methylation identified in bulk cortex and FANS-isolated nuclei populations from neuron-enriched and neuron-depleted cells, related to Figure 1 and Figure 4. Shown for the 42,114 bulk cortex dDMPs also tested in FANS-isolated populations is the correlation of developmental changes between **A)** bulk fetal cortex and SATB2+ (neuron-enriched) nuclei, **B)** bulk cortex and SATB2- (neuron-depleted) nuclei and **C)** SATB2+ (neuron-enriched) and SATB2- (neuron-depleted) nuclei. Effect size represents the percentage change in DNA methylation per post-conception week. Solid line = regression line. Dotted line = y=x.

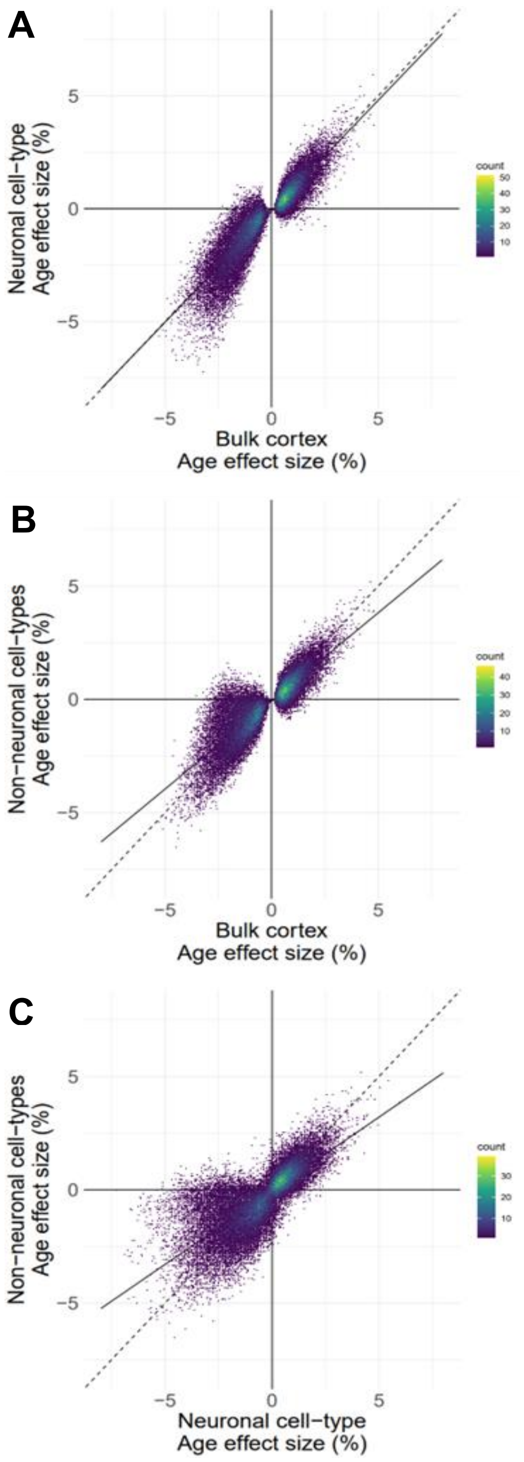

Figure S18 – Overlap of developmental changes in DNA methylation identified in SATB2+ (“neuronal”) and SATB2- (“non-neuronal”) nuclei isolated from the fetal cortex, related to Figure 4. Of the 1,872 SATB2+ and 820 SATB2- dDMPs, 272 are consistent between cell-types ($p < 9x10^{-8}$), all of which are concordant in direction.

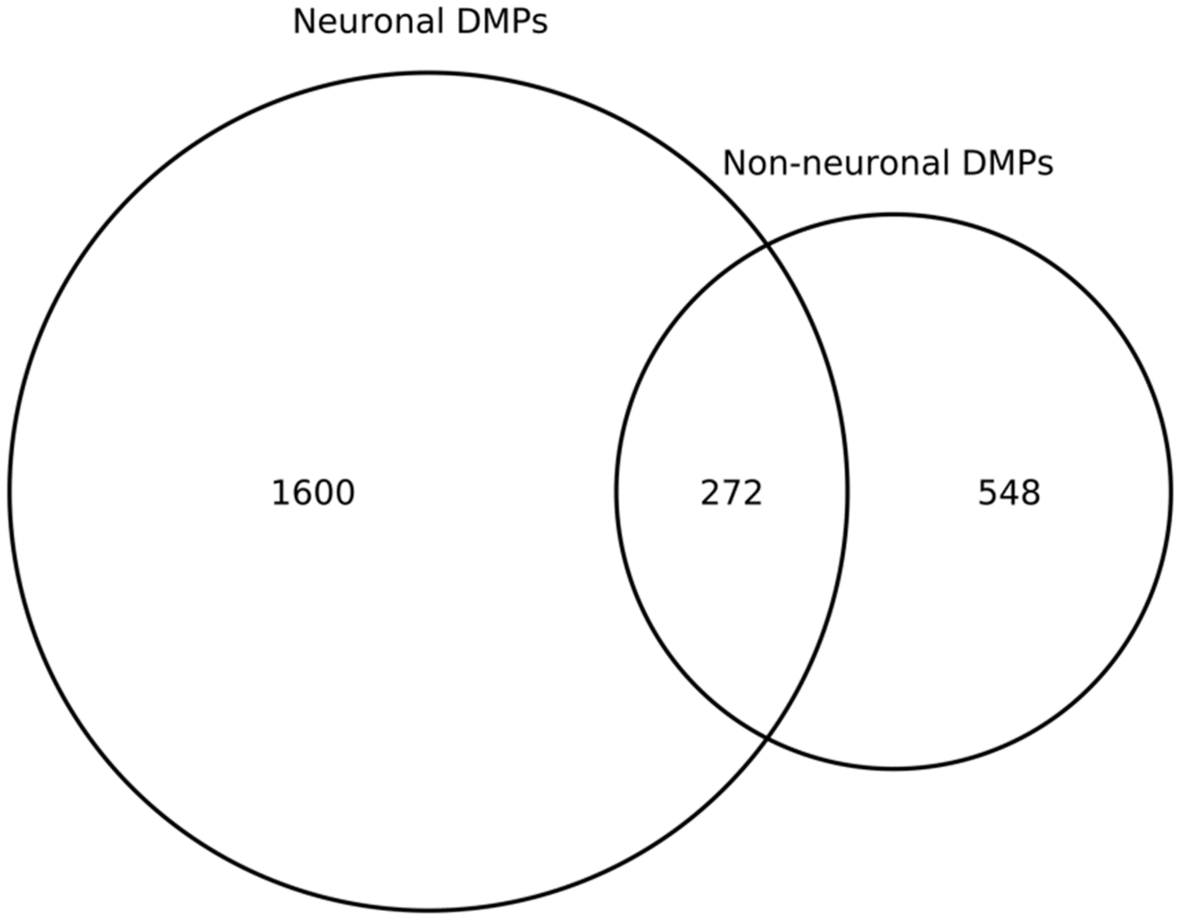

Figure S19 – Overlap of dDMPs identified in bulk fetal cortex and purified nuclei populations, related to Figure 1 and Figure 4. Overlap of the 42,114 bulk cortex dDMPs ($p < 9x10^{-8}$) also tested in our FANS-isolated samples with the 4,088 SATB2+ dDMPs ($p < 1.19x10^{-6}$) and 1,820 SATB2- dDMPs ($p < 1.19x10^{-6}$).

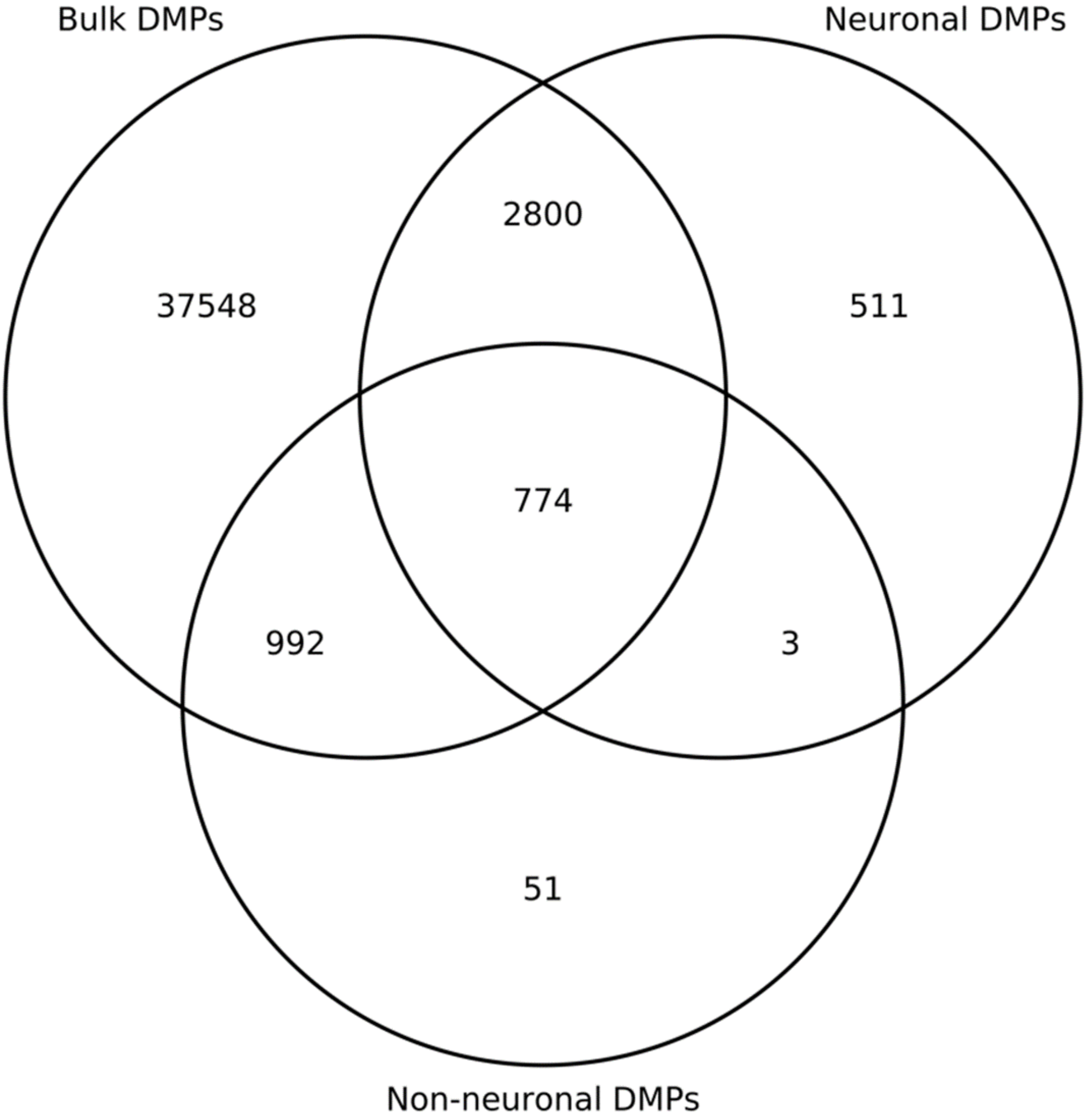

Figure S20 – Many developmental changes in DNA methylation identified the bulk fetal cortex reflect changes that are specific to SATB2+ or SATB2- nuclei, related to Figure 4. Shown are examples of bulk cortex dDMPs that are characterized by **A)** developmental changes only in SATB2+ nuclei, **B)** developmental changes only in SATB2- nuclei and **C-D)** opposite developmental changes in SATB2+ and SATB2- nuclei. **B)** cg00173446 (annotated to *CDK17*) shows a decrease in DNA methylation specific to the SATB2- population, consistent with reports of neuron-specific *CDK17* expression during cortex development [9]. **C)** cg01701649 (annotated to *N4BP1*, a gene involved in mediating neurogenesis in the developing cortex [10]) shows diverging DNA methylation levels between SATB2+ (reducing DNA methylation) and SATB2- (increasing DNA methylation) nuclei. **D)** In contrast, cg19462364 (annotated to *BLM*, a gene involved in regulating neuronal apoptosis during brain development [11]), is characterised by hypermethylation of the SATB2+ population and hypomethylation of SATB2- nuclei. Shaded region indicates 95% confidence interval.

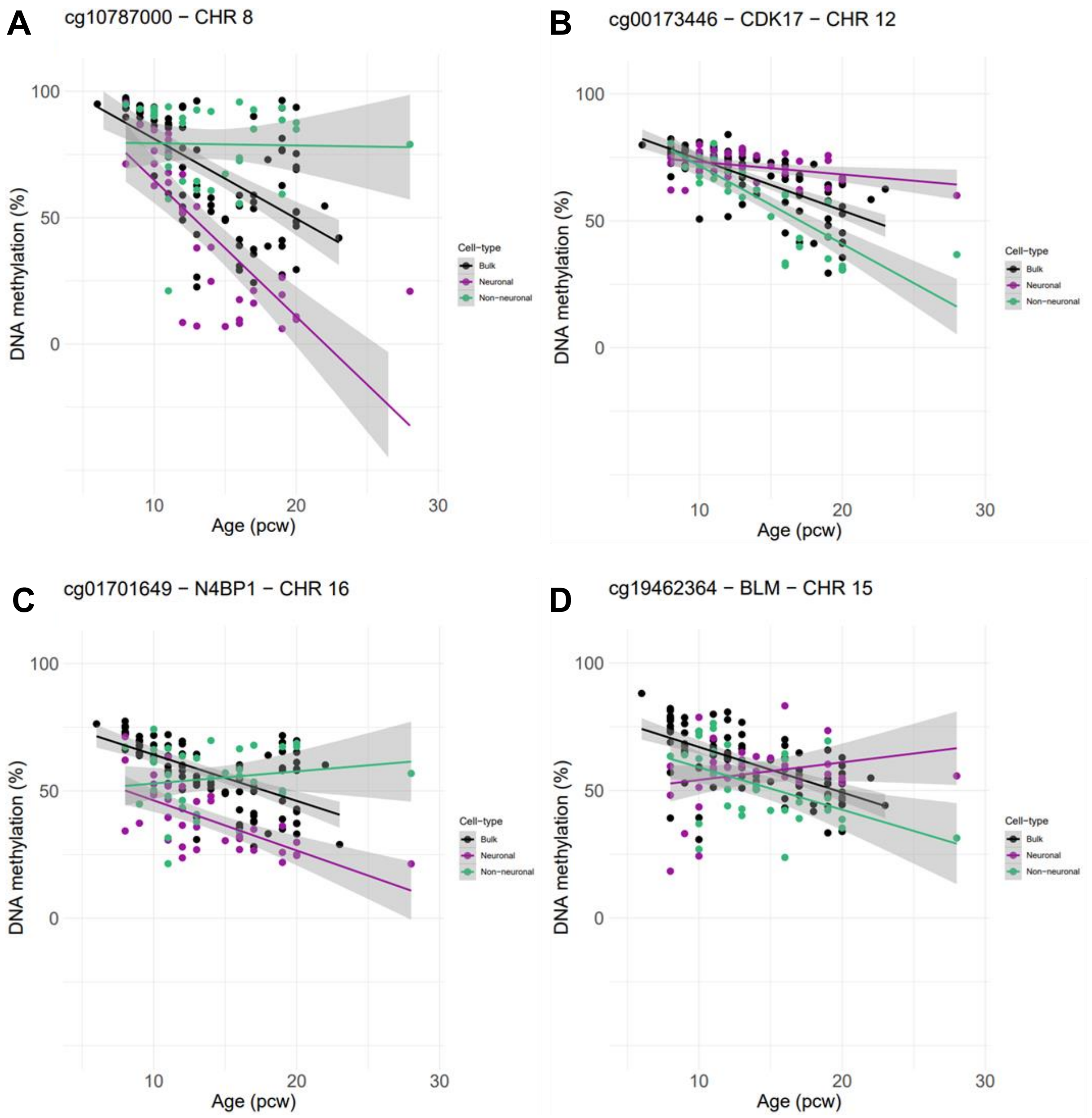

Figure S21 – Identification of cell-type specific changes in DNA methylation in human cortex development, related to Figure 4. Comparison of age effect sizes for **A)** dDMPs identified in either SATB2+ neuronal or SATB2- non-neuronal nuclei (n = 2,420, corr = 0.564), **B)** SATB2+ neuronal dDMPs (n = 1,872, corr = 0.634) and **C)** SATB2- non-neuronal dDMPs (n = 820, corr = 0.873). Effect size represents the change in DNA methylation (%) per post-conception week. The results show that developmental changes identified in non-neuronal nuclei are broadly reflected in SATB2+ neuronal nuclei, but a large proportion of neuronal dDMPs are cell-type-specific.

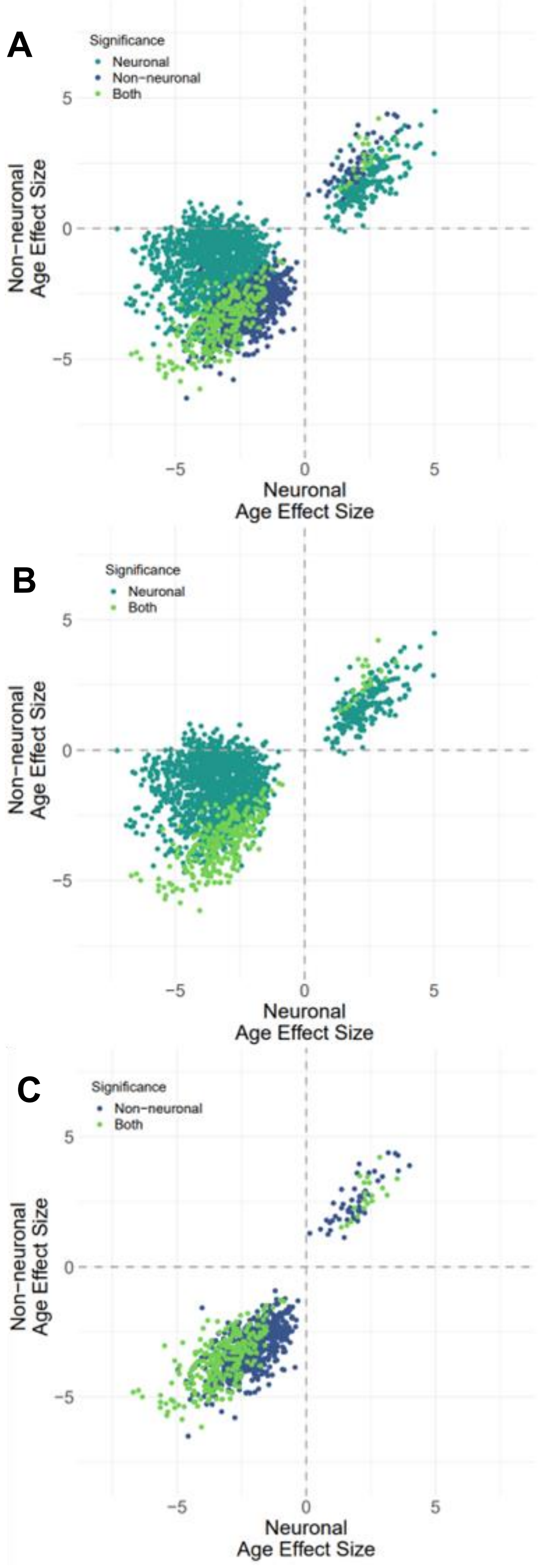

Figure S22 – Examples of sites characterized by cell-type-specific developmental changes in DNA methylation in the fetal cortex, related to Figure 4. A) cg12430457 (annotated to *SYT1*, a gene involved in synaptic vesicle exocytosis [12]) becomes developmentally hypomethylated in SATB2+ neuronal nuclei but not SATB2- nuclei. **B)** cg09165170 (annotated to *PEX14*, a gene that encodes a peroxisomal protein crucial for import of cargo into peroxisomes which are critical in oligodendrocytes for the maintenance of myelination [13]) becomes developmentally hypermethylated in SATB2+ neuronal nuclei but not SATB2- nuclei. **C)** cg13609939 (annotated to *OAT*, a gene involved in the glutamate metabolic pathway [14,15]) shows a greater rate of hypomethylation in SATB2- nuclei compared to SATB2+ neuronal nuclei. **D)** cg23699648 (annotated to *GJA1*, encoding a connexin protein that plays an important role in neural development [16]) shows a greater rate of hypermethylation in SATB2+ neuronal nuclei compared to SATB2- nuclei. A full list of sites showing cell-type-specific developmental changes in DNA methylation in the fetal cortex is given in **Table S11**.

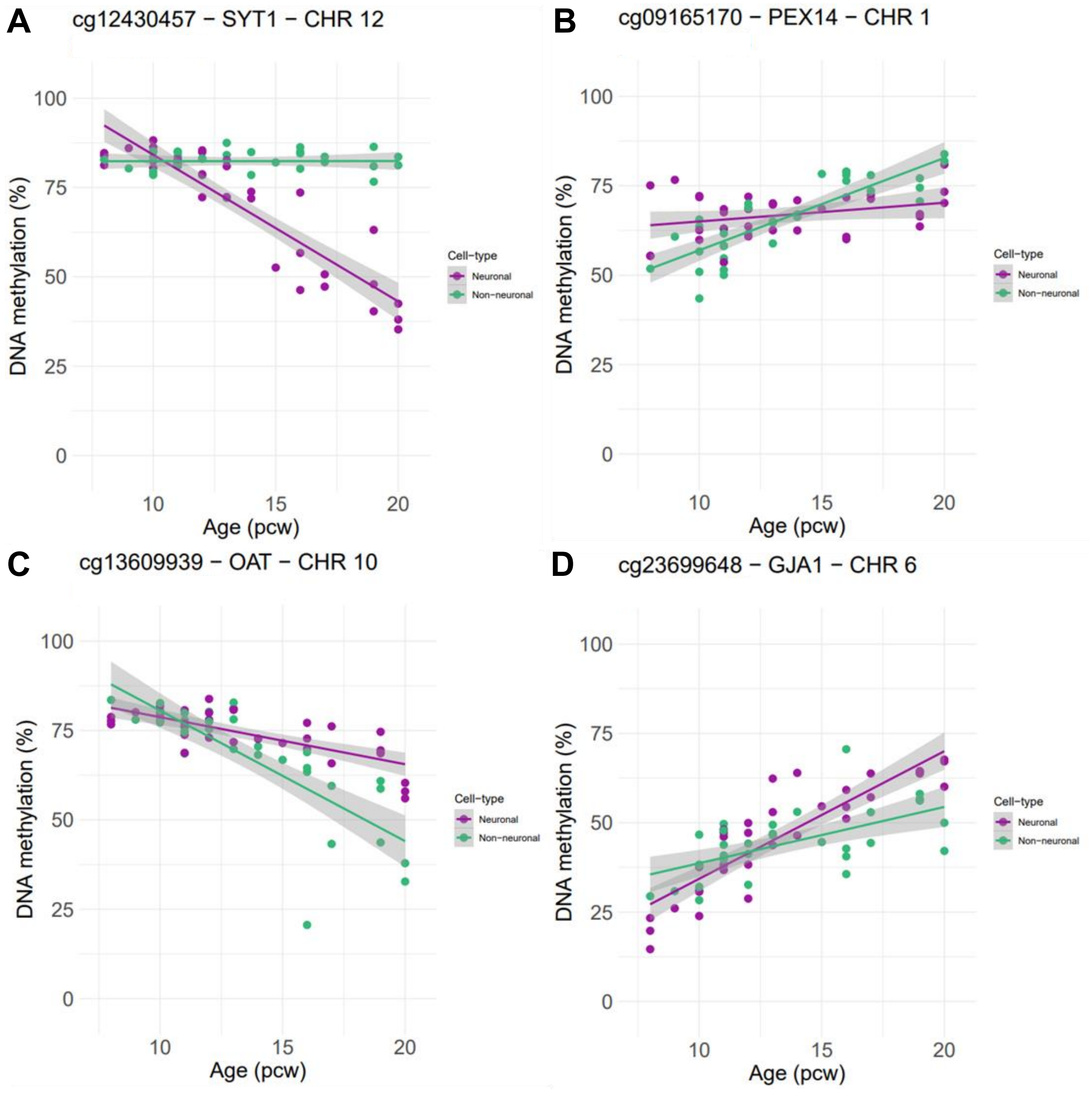

Figure S23 – Enrichment of cell-type-specific dDMPs in regions of open chromatin identified in relevant cell-types by scATAC-seq, related to Figure 4. Relative effect size from a logistic regression analysis testing for an enrichment of dDMPs within scATAC-seq peaks obtained from a published analysis of 54 human fetal cell-types [4]. **A)** Enrichment of SATB2+-specific autosomal dDMPs (n = 1,596) within cell-type-specific ATAC-seq peaks. Excitatory neurons demonstrate the single clearest enrichment (see also **Table S12**). **B)** In contrast, intersecting SATB2--specific autosomal dDMPs (n=548) with cell-type-specific ATAC-seq peaks shows a clear enrichment for astrocytes (see also **Table S13**).

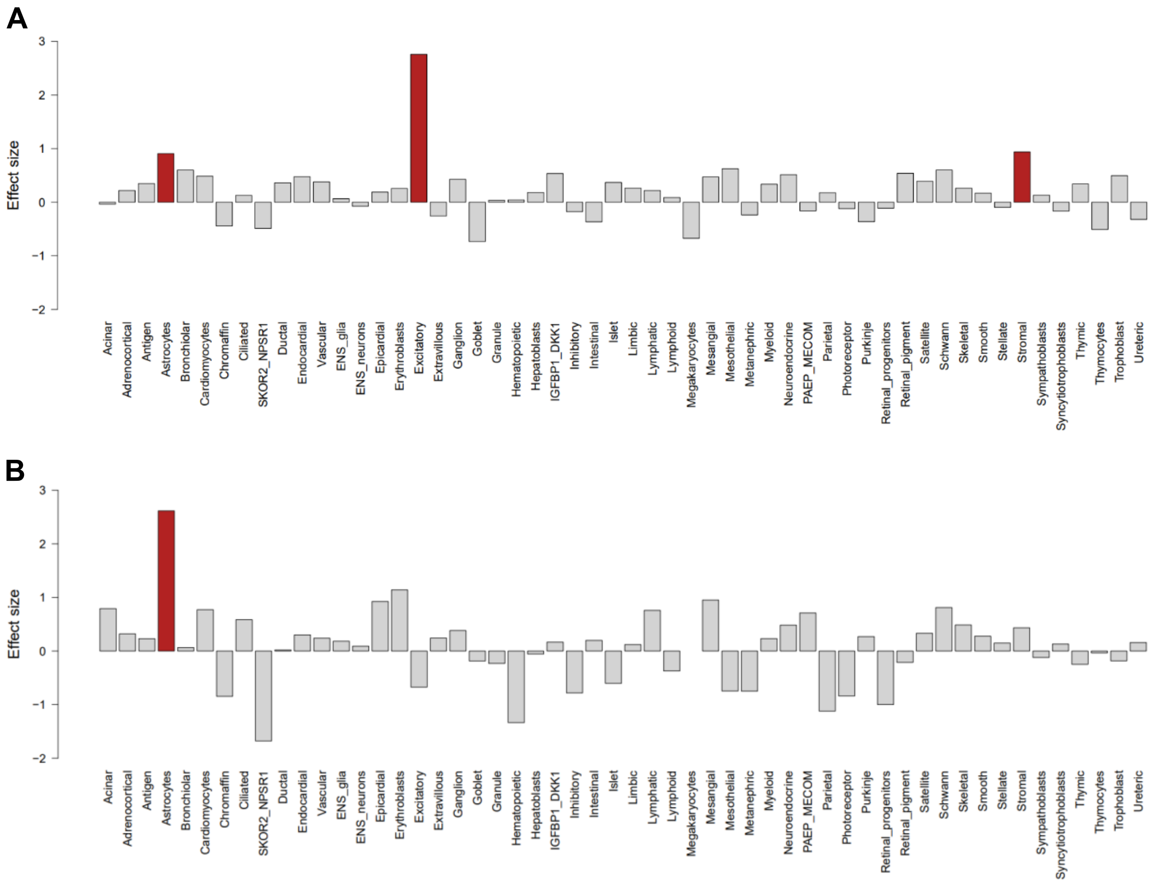

Figure S24 – Proportion of bulk fetal cortex dDMPs annotated to autism (SFARI) and schizophrenia (SCHEMA) genes, related to STAR Methods. A) Percentage of genes that contain at least one dDMP across four gene lists: i) all 26,728 genes annotated to sites on the array (gray), ii) the 233 SFARI genes (blue), iii) the 32 SCHEMA genes (green) and iv) the 259 combined unique SFARI and SCHEMA genes (purple). **B)** Percentage of all sites annotated to each of the gene lists that is a dDMP.

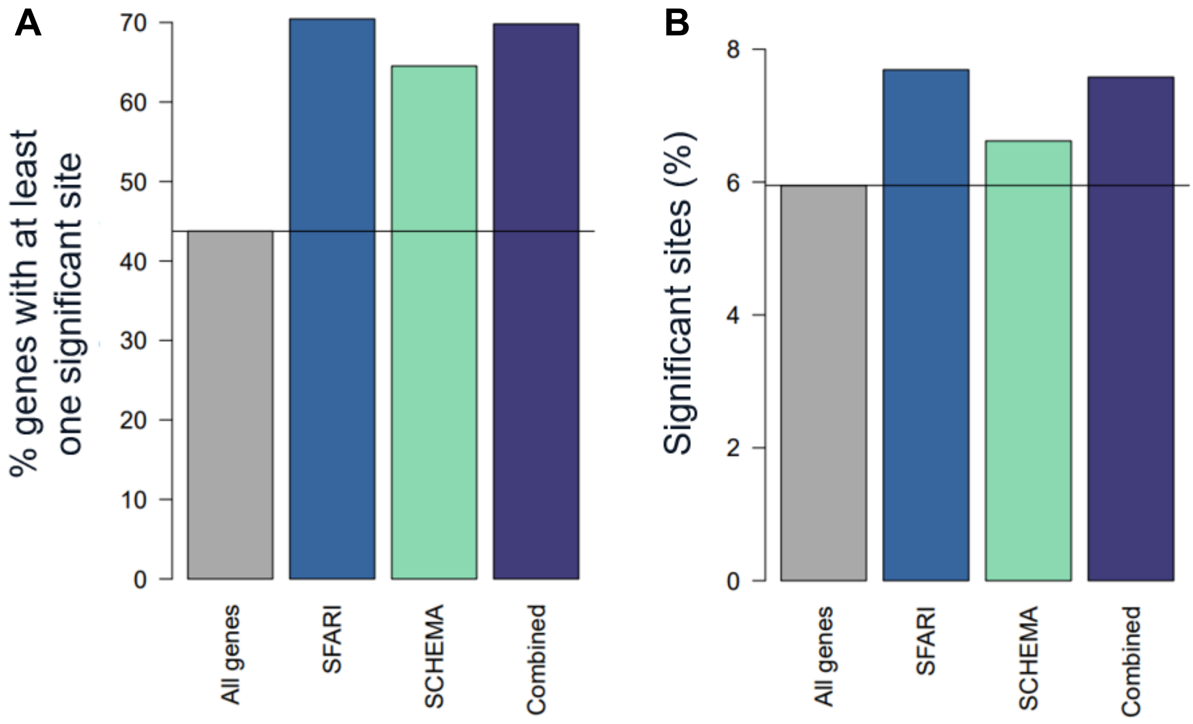

Figure S25 – Proportion of neuronal and non-neuronal dDMPs annotated to autism (SFARI) and schizophrenia (SCHEMA) genes, related to STAR Methods. A) Percentage of genes that contain at least one SATB2+ neuronal dDMP across four gene lists: i) all 26,455 genes annotated to sites on the array (grey), ii) the 233 SFARI genes (blue), and iii) the 32 SCHEMA genes (green) and iv) the 259 combined SFARI and SCHEMA genes (purple). **B)** Percentage of all sites annotated to each gene list that is a SATB2+ neuronal dDMP. **C)** Percentage of genes that contain at least one SATB2- dDMP across each of the four gene lists. **D)** Percentage of all sites annotated to each gene list that is a SATB2- dDMP.

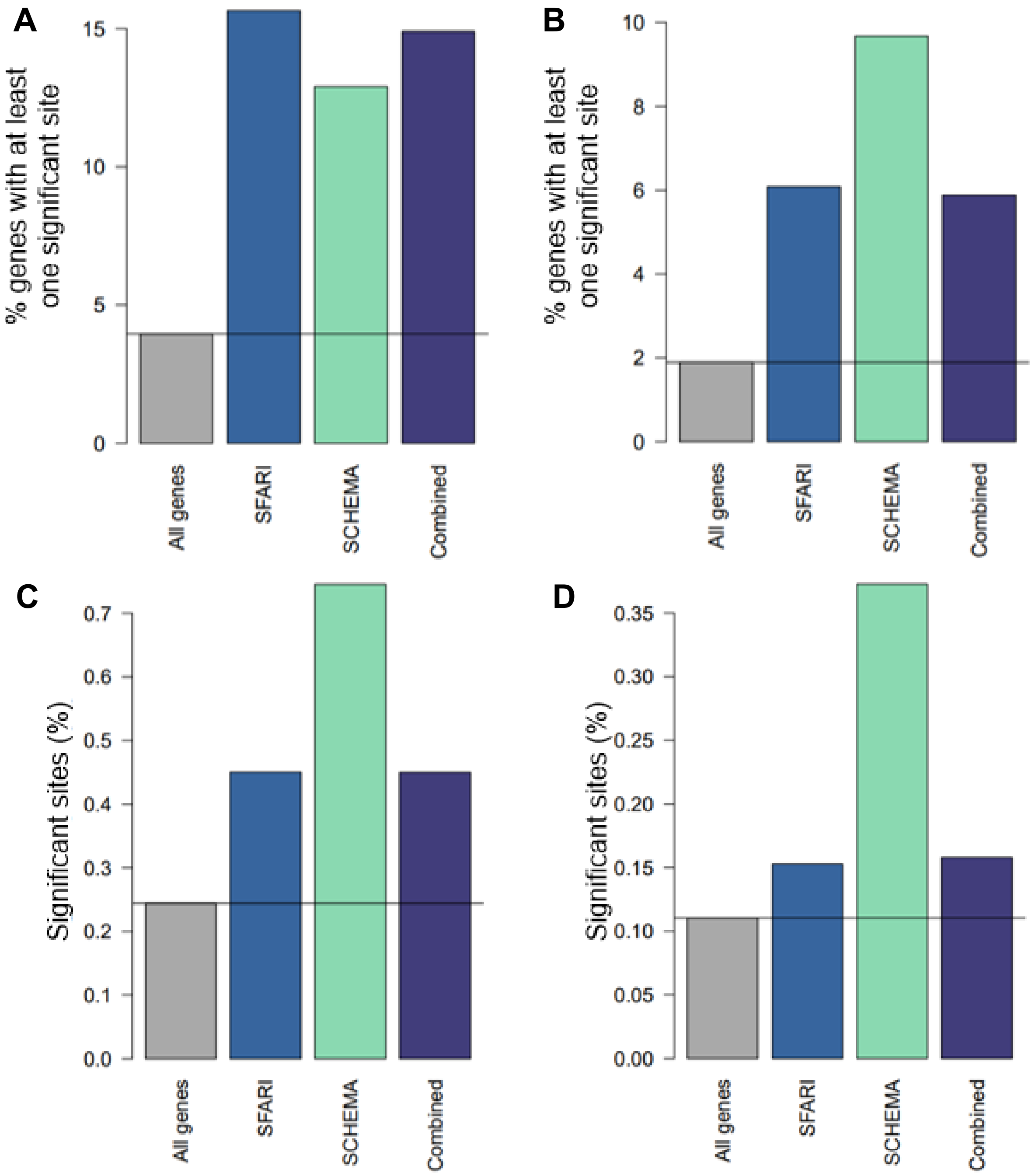

Figure S26 – Enrichment of dDMPs for autism (SFARI) and schizophrenia (SCHEMA) genes is strongest for highest-ranked dDMPs, related to STAR Methods.
Shown are the odds ratios for the enrichment of genes annotated to **A)** bulk cortex dDMPs and **B)** SATB2+ neuron dDMPs amongst i) combined SFARI and SCHEMA genes (n=259), ii) SFARI genes (n=233), and iii) SCHEMA genes (n=32). Enrichment was tested on dDMPs (ranked most significant to least significant) partitioned into bins. For bulk cortex we sequentially increased the number of dDMPs tested for enrichment by 10,000 sites. For SATB2+ neurons we sequentially increased the number of dDMPs tested for enrichment by 200 sites. 'All dDMPs' represents the odds ratio for the complete set of dDMPs. There was an insufficient number of SATB2- dDMPs (n=820) to perform a sequential analysis on these sites.

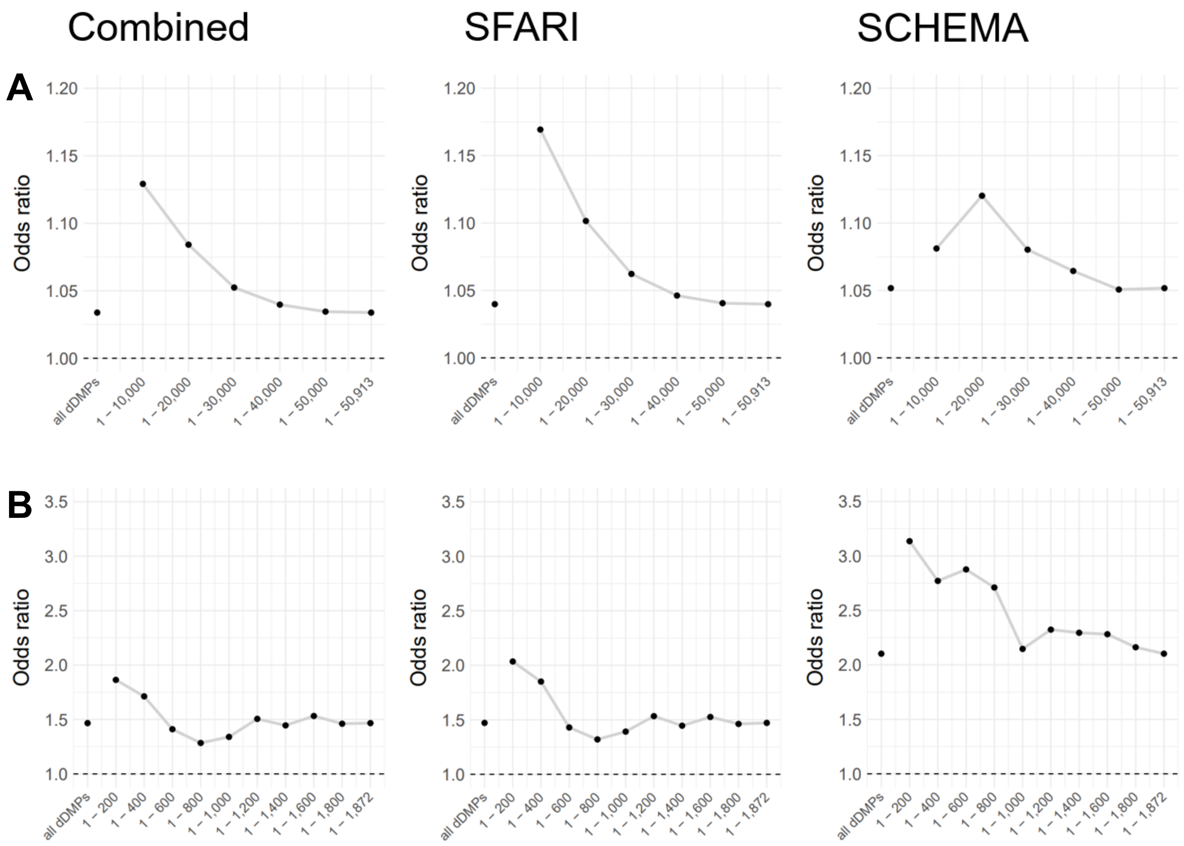

Figure S27 – MAGMA gene set enrichment, related to STAR Methods. Enrichment of genes annotated to bulk cortex, neuronal (SATB2+) and non-neuronal (SATB2-) dDMPs for common variants associated with **A)** autism and **B)** schizophrenia using MAGMA [17]. Circles indicate enrichment effect size +/- 95% confidence interval. p = unadjusted p-value.

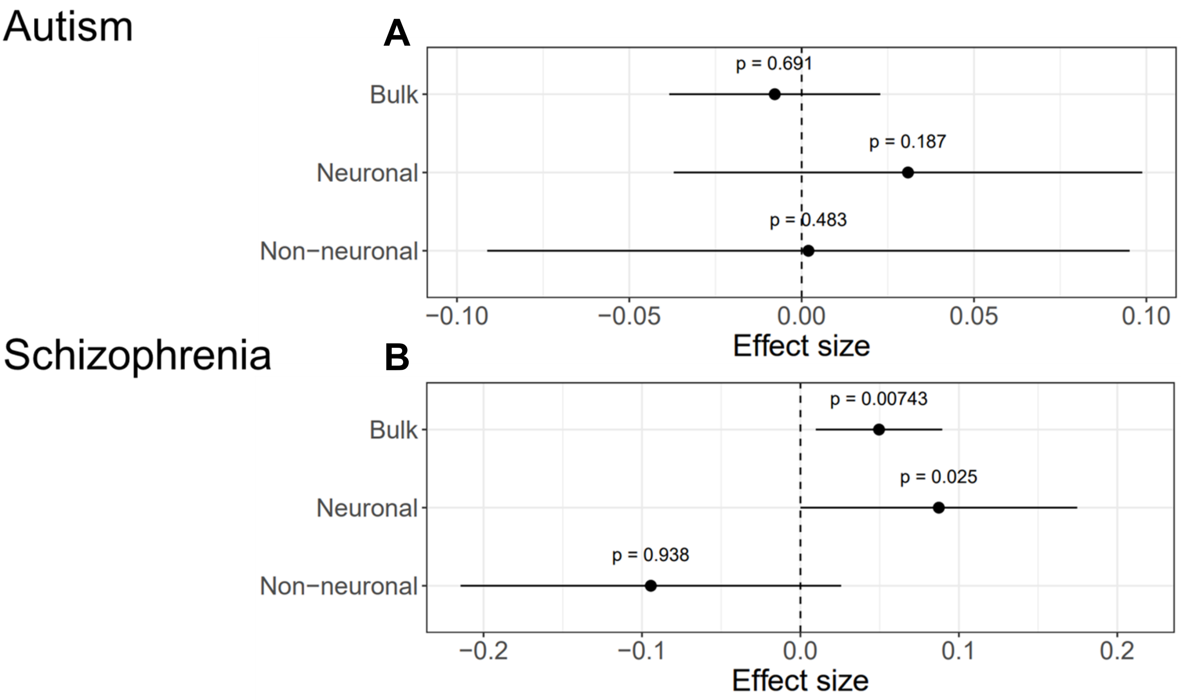

Figure S28 – Log-likelihood ratio to refine nonlinear sites, related to STAR Methods. Shown is timescale against the log-likelihood ratios (LLRs) of **A)** the Matern 5/2 (nonlinear) vs constant kernel and **B)** Matern 5/2 vs linear kernel, for the 175,419 nonlinear sites identified from the Gaussian process model as having the greatest marginal log-likelihood for the Matern 5/2 kernel. Sites with LLR < 2 and timescale < 10 were excluded, leaving 73,638 high-confidence nonlinear sites with biologically meaningful timescales between 10.0 and 106. Color bar indicates number of nonlinear sites per bin.

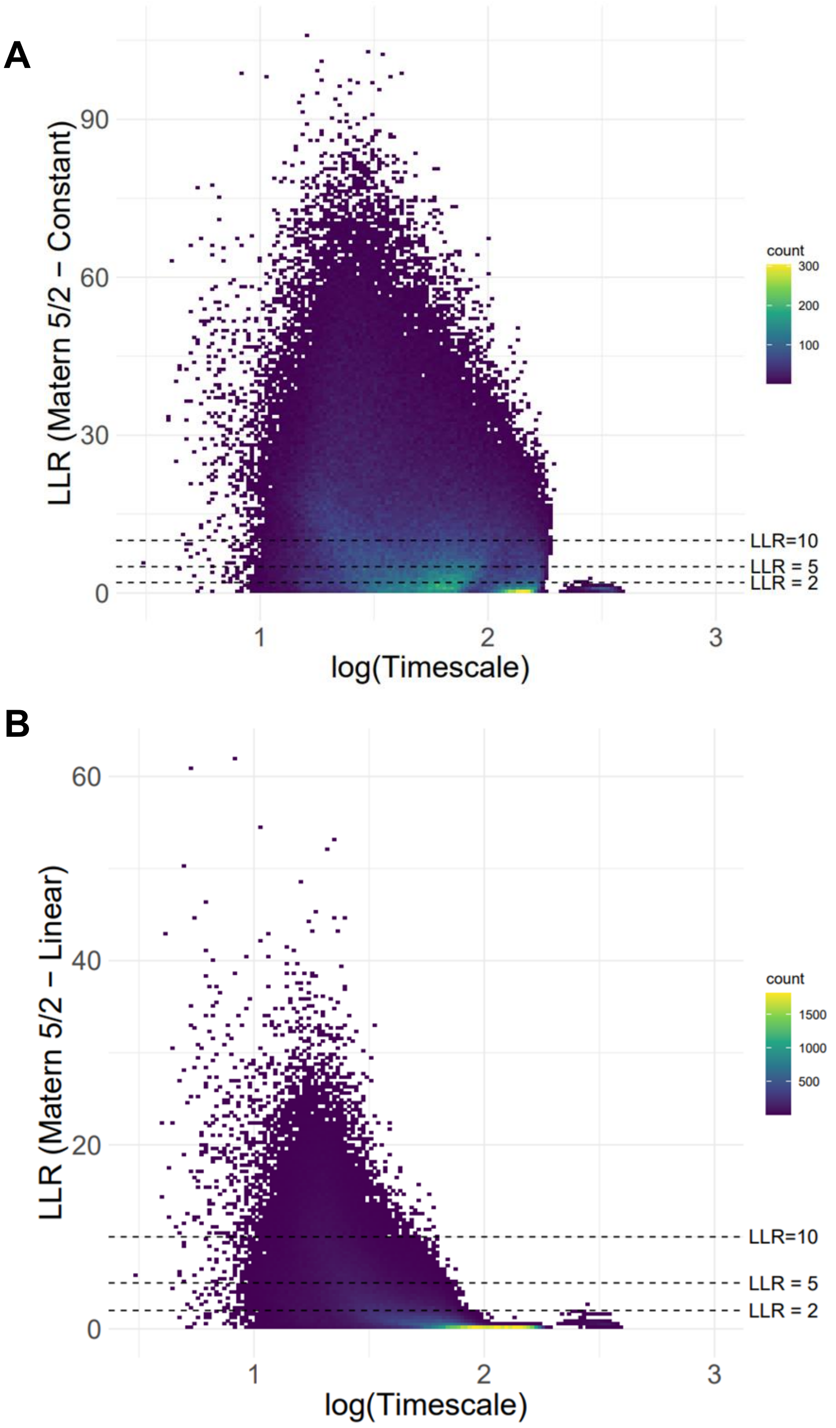

Figure S29 – Nonlinear DNA methylation sites most representative of the module eigengene ("hub sites") , related to STAR Methods and Figure 2. For each of the six nonlinear modules, shown are the top three DNA methylation sites most highly correlated to the module eigengene.

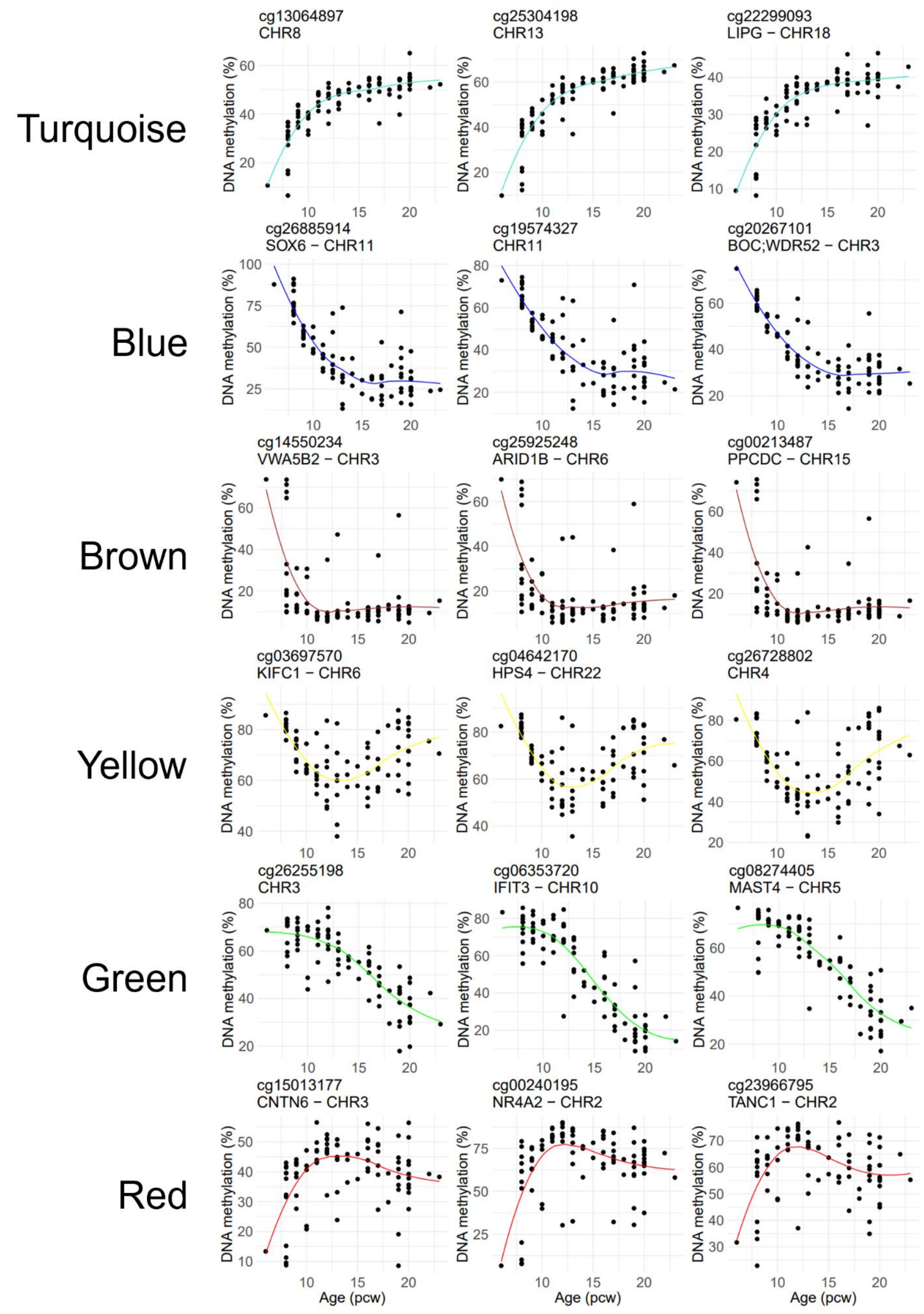

REFERENCES

1. Steg, L.C., Shireby, G.L., Imm, J., Davies, J.P., Franklin, A., Flynn, R., Namboori, S.C., Bhinge, A., Jeffries, A.R., Burrage, J., et al. (2021). Novel epigenetic clock for fetal brain development predicts prenatal age for cellular stem cell models and derived neurons. Mol Brain *14*, 1–11. https://doi.org/https://doi.org/10.1186/s13041-021-00810-w.
2. Spiers, H., Hannon, E., Schalkwyk, L.C., Smith, R., Wong, C.C.Y., O'Donovan, M.C., Bray, N.J., and Mill, J. (2015). Methylomic trajectories across human fetal brain development. Genome Res *25*, 338–352. https://doi.org/10.1101/GR.180273.114.
3. Numata, S., Ye, T., Hyde, T.M., Guitart-Navarro, X., Tao, R., Wininger, M., Colantuoni, C., Weinberger, D.R., Kleinman, J.E., and Lipska, B.K. (2012). DNA Methylation Signatures in Development and Aging of the Human Prefrontal Cortex. Am J Hum Genet *90*, 260. https://doi.org/10.1016/J.AJHG.2011.12.020.
4. Domcke, S., Hill, A.J., Daza, R.M., Cao, J., O'Day, D.R., Pliner, H.A., Aldinger, K.A., Pokholok, D., Zhang, F., Milbank, J.H., et al. (2020). A human cell atlas of fetal chromatin accessibility. Science (1979) *370*. https://doi.org/https://doi.org/10.1126/science.aba7612.
5. Horvath, S. (2013). DNA methylation age of human tissues and cell types. Genome Biol *14*, 1–20. https://doi.org/https://doi.org/10.1186/gb-2013-14-10-r115.
6. Shireby, G.L., Davies, J.P., Francis, P.T., Burrage, J., Walker, E.M., Neilson, G.W.A., Dahir, A., Thomas, A.J., Love, S., Smith, R.G., et al. (2020). Recalibrating the epigenetic clock: implications for assessing biological age in the human cortex. Brain *143*, 3763–3775. https://doi.org/10.1093/BRAIN/AWAA334.
7. Jaffe, A.E., Straub, R.E., Shin, J.H., Tao, R., Gao, Y., Collado-Torres, L., Kam-Thong, T., Xi, H.S., Quan, J., Chen, Q., et al. (2018). Developmental and genetic regulation of the human cortex transcriptome illuminate schizophrenia pathogenesis. Nat Neurosci *21*, 1117. https://doi.org/10.1038/S41593-018-0197-Y.
8. Kim, M., Park, Y.K., Kang, T.W., Lee, S.H., Rhee, Y.H., Park, J.L., Kim, H.J., Lee, D., Lee, D., Kim, S.Y., et al. (2014). Dynamic changes in DNA methylation and hydroxymethylation when hES cells undergo differentiation toward a neuronal lineage. Hum Mol Genet *23*, 657–667. https://doi.org/10.1093/HMG/DDT453.
9. Hirose, T., Tamaru, T., Okumura, N., Nagai, K., and Okada, M. (1997). PCTAIRE 2, a Cdc2-related serine/threonine kinase, is predominantly expressed in terminally differentiated neurons. Eur J Biochem *249*, 481–488. https://doi.org/10.1111/J.1432-1033.1997.T01-1-00481.X.
10. Ma, Z., Zeng, Y., Wang, M., Liu, W., Zhou, J., Wu, C., Hou, L., Yin, B., Qiang, B., Shu, P., et al. (2023). N4BP1 mediates RAM domain-dependent notch signaling turnover during neocortical development . EMBO J *42*. https://doi.org/10.15252/EMBJ.2022113383.
11. Liu, W.W., Chen, S.Y., Cheng, C.H., Cheng, H.J., and Huang, P.H. (2014). Blm-s, a BH3-only protein enriched in postmitotic immature neurons, is transcriptionally upregulated by p53 during DNA damage. Cell Rep *9*, 166–179. https://doi.org/10.1016/j.celrep.2014.08.050.
12. Riggs, E., Shakkour, Z., Anderson, C.L., and Carney, P.R. (2022). SYT1-Associated Neurodevelopmental Disorder: A Narrative Review. Children (Basel) *9*. https://doi.org/10.3390/CHILDREN9101439.

13. Bottelbergs, A., Verheijden, S., Hulshagen, L., Gutmann, D.H., Goebbels, S., Nave, K.A., Kassmann, C., and Baes, M. (2010). Axonal integrity in the absence of functional peroxisomes from projection neurons and astrocytes. Glia *58*, 1532–1543. https://doi.org/10.1002/GLIA.21027.
14. Rumping, L., Vringer, E., Houwen, R.H.J., van Hasselt, P.M., Jans, J.J.M., and Verhoeven-Duif, N.M. (2020). Inborn errors of enzymes in glutamate metabolism. J Inherit Metab Dis *43*, 200–215. https://doi.org/10.1002/JIMD.12180.
15. Ginguay, A., Cynober, L., Curis, E., and Nicolis, I. (2017). Ornithine Aminotransferase, an Important Glutamate-Metabolizing Enzyme at the Crossroads of Multiple Metabolic Pathways. Biology (Basel) *6*, 18. https://doi.org/10.3390/BIOLOGY6010018.
16. Cao, J.W., Liu, L.Y., and Yu, Y.C. (2023). Gap junctions regulate the development of neural circuits in the neocortex. Curr Opin Neurobiol *81*, 102735. https://doi.org/10.1016/J.CONB.2023.102735.
17. de Leeuw, C.A., Mooij, J.M., Heskes, T., and Posthuma, D. (2015). MAGMA: Generalized Gene-Set Analysis of GWAS Data. PLoS Comput Biol *11*, e1004219. https://doi.org/10.1371/JOURNAL.PCBI.1004219.
